# Supplementary material for: Conceptual coherence but methodological mayhem: A systematic review of absolute pitch phenotyping
Source: Behav Res Methods. 2025 Jan 21;57(2):61. doi: 10.3758/s13428-024-02577-z (PMC11750914; doi:10.3758/s13428-024-02577-z)

Conceptual Coherence but Methodological Mayhem: A Systematic Review of Absolute Pitch Phenotyping

Jane E. Bairnsfather^1*^, Miriam A. Mosing^1,2,3^, Margaret S. Osborne^1,4^, & Sarah J. Wilson^1^

^1^Melbourne School of Psychological Sciences, University of Melbourne, Melbourne, Australia

^2^Department of Neuroscience, Karolinska Institutet, Stockholm, Sweden

^3^Department of Cognitive Neuropsychology, Max Planck Institute for Empirical Aesthetics, Frankfurt Am Main, Germany

^4^Melbourne Conservatorium of Music, University of Melbourne, Melbourne, Australia

*Corresponding author

Melbourne School of Psychological Sciences, University of Melbourne, Melbourne, Australia. Email: [jbairnsfathe@student.unimelb.edu.au](mailto:jbairnsfathe@student.unimelb.edu.au)

Abstract

Despite extensive research on absolute pitch (AP), there remains no gold-standard task to measure its presence or extent. This systematic review investigated the methods of pitch-naming tasks for the classification of individuals with AP and examined how our understanding of the AP phenotype is affected by variability in the tasks used to measure it. Data extracted from 160 studies (*N* = 23,221 participants) included (i) the definition of AP, (ii) task characteristics, (iii) scoring method, and (iv) participant scores. While there was near-universal agreement (99%) in the conceptual definition of AP, task characteristics such as stimulus range and timbre varied greatly. Ninety-five studies (59%) specified a pitch-naming accuracy threshold for AP classification, which ranged from 20-100% (mean = 77%, *SD* = 20), with additional variability introduced by 31 studies that assigned credit to semitone errors. When examining participants’ performance rather than predetermined thresholds, mean task accuracy (not including semitone errors) was 85.9% (*SD* = 10.8) for AP participants and 17.0% (*SD* = 10.5) for non-AP participants. This review shows that characterisation of the AP phenotype varies based on methodological choices in tasks and scoring, limiting the generalisability of individual studies. To promote a more coherent approach to AP phenotyping, recommendations about the characteristics of a gold-standard pitch-naming task are provided based on the review findings. Future work should also use data-driven techniques to characterise phenotypic variability to support the development of a taxonomy of AP phenotypes to advance our understanding of its mechanisms and genetic basis.

Keywords: absolute pitch, phenotype, heritability, methods

Introduction

Absolute pitch (AP) is the uncommon ability to identify and label isolated musical pitches in the absence of a reference tone. It contrasts to relative pitch, the ability to use relationships between pitches in a musical context. While relative pitch is a necessary skill for musicians and can be developed through practice (Miyazaki et al., 2018), AP is thought to be present in only a small percentage of musicians, although estimates vary widely from < 1% to 65% across studies; Deutsch et al., 2006; Leite et al., 2016; Miyazaki et al., 2012; Miyazaki et al., 2018) and in most studies cannot be reliably trained (Bittrich et al., 2015; Brady, 1970; Cuddy, 1968, 1970; Gregersen et al., 1999; Leite et al., 2016; Profita & Bidder, 1988; Sakakibara, 2014; although see Van Hedger et al., 2019 for evidence of the skill acquisition theory of AP). Although not necessary for musicianship, individuals with AP describe it as integral to their perception of the auditory world, with one AP musician musing that “people who did not have absolute pitch must be tone deaf to a certain extent” (Boggs, 1907, p. 204). AP can be beneficial (e.g., singing in-tune unaccompanied), but can also be a hindrance (e.g., difficulty listening to music using non-standard tuning; West Marvin et al., 2020).

AP is of interest due to its rarity, its discreteness as a behavioural trait, and the mechanisms by which such an unusual ability is acquired and maintained. A substantial body of literature has explored AP regarding environmental and heritable predisposing factors (Baharloo et al., 1998; Brown et al., 2002; Deutsch et al., 2009; Levitin & Zatorre, 2003; Miyazaki et al., 2012; Vanzella & Schellenberg, 2010; Vitouch, 2003; Wilson et al., 2012), its relationship to other musical skills (Dohn et al., 2014; Dooley & Deutsch, 2010, 2011; Jiang et al., 2010; Miyazaki, 2004b; West Marvin et al., 2020; Ziv & Radin, 2014), cognitive correlates (Benassi-Werke et al., 2012; Brancucci, di Nuzzo, et al., 2009; Burnham et al., 2015; Deutsch & Dooley, 2013; Greber & Jäncke, 2020; Hou et al., 2021; Hou et al., 2014; Hutka & Alain, 2015; Wenhart & Altenmüller, 2019), and neuroanatomical markers (Bermudez et al., 2009; Brauchli et al., 2019; Burkhard et al., 2019; Burkhard et al., 2020; Dohn et al., 2015; Elmer et al., 2015; Greber et al., 2018; Jäncke et al., 2012; Kim & Knösche, 2016; Leipold, Brauchli, et al., 2019; Maeshima et al., 2018; McKetton et al., 2019; Schulze et al., 2013; Wengenroth et al., 2014; Wilson et al., 2009).

Among the earliest AP research was the suggestion that AP is at least partly heritable, due to its appearance in early childhood without deliberate practice, and its tendency to accrue in musical families (Bachem, 1940, 1948; Boggs, 1907; Seashore, 1939, 1940). Bachem (1940), for example, observed that 39% of AP possessors in a sample of 103 had relatives with AP. Subsequent findings have also supported a heritable component, including proposed models of inheritance and chromosomal loci of interest (Baharloo et al., 1998; Baharloo et al., 2000; Bairnsfather, Ullén, et al., 2022; Gregersen et al., 1999; Gregersen et al., 2013; Profita & Bidder, 1988; Theusch et al., 2009; Theusch & Gitschier, 2011). While this is certainly suggestive of genetic variants for AP, further progress in this area has been hindered by a lack of consensus regarding the AP phenotype.

AP is of particular relevance in the study of individual differences. Exploration of trait heritability has, over time, moved from classical twin modelling (Polderman et al., 2015) to genome-wide association studies (Abdellaoui & Verweij, 2021), with both approaches showing that behavioural traits are broadly heritable. AP is a useful model to explore this heritability, as it is a rather discrete behavioural trait which has been documented to run in families. As such, it is important to accurately phenotype AP, both due to the intrinsic fascination of the ability itself, and for its potential applicability to broader behavioural genetics research.

Phenotyping refers to efforts to classify observable characteristics in behavioural traits and syndromes, and is a necessary foundation on which to build an understanding of a trait’s biological mechanisms and genetic influences. The AP phenotype, conceptually described as pitch identification without a reference, is typically measured by participant performance on a behavioural task, most commonly pitch-naming (Takeuchi & Hulse, 1993). In such a task, participants are presented with a series of auditory pitches and are required to identify their musical labels (e.g., G, B flat). Individuals with AP should be able to complete this task effortlessly and with a high level of accuracy, in the absence of external aids. Those without any AP ability can only guess their responses and are therefore expected to perform around chance level (1/12 or 8.3% as there are 12 chroma, or pitch classes, in the Western musical scale). While this appears to be a clear phenotypic distinction, multiple factors make the delineation of an AP phenotype more complex.

First, some individuals are able to identify pitches above chance, but below typical AP-levels of accuracy. These individuals are variously referred to as possessing quasi-AP (QAP, (Bachem, 1937), partial AP or white-key note AP (Miyazaki, 2004a), raising the idea of multiple phenotypes. QAP possessors are thought to be able to identify some, but not all chroma, and may be able to use relative pitch strategies to identify unknown chroma from an internal reference of their preferred chroma (Bairnsfather, Osborne et al., 2022; Wilson et al., 2009). QAP has been considered in relatively few investigations of AP. Thus, it has not been established whether it can be reliably distinguished from AP using a pitch-naming accuracy threshold (as in Aruffo et al., 2014; Bairnsfather, Osborne, et al., 2022; Chavarria-Soley, 2016; Leipold, Oderbolz, et al., 2019; Wilson et al., 2012; Wilson et al., 2009), or whether AP and QAP should be considered along a pitch-naming continuum (for a recent discussion, see Van Hedger et al., 2020).

Second, even when intermediate pitch-naming performance is not explicitly included, accuracy thresholds for AP possession vary across studies (e.g., 90%, Aruffo et al., 2014; 68%, Athos et al., 2007). While AP possessors are expected to be highly accurate, the precise level of performance required has not been agreed upon. Thresholds can also be rendered less conservative by the inclusion of credit for small errors. As AP possessors age, they may report a shift in the accuracy of their internal pitch templates, prompting them to make occasional pitch-naming errors (Athos et al., 2007). Some researchers choose to compensate for this by assigning partial or full credit to errors within a semitone (a distance of one chroma) of the correct response for all participants or those within specific age ranges (Athos et al., 2007).

Aside from scoring and threshold concerns, characterisation of an AP phenotype is further hindered by the lack of a gold-standard pitch-naming task. One of the most salient task characteristics is the timbre of the presented stimuli. Although some individuals with AP can identify a predominant pitch in environmental sounds, such as a spoken voice or car engine (Heaton et al., 2008), studies generally use either pure tones (e.g., Burkhard et al., 2019) or synthesised or recorded instrumental tones (e.g., Deutsch et al., 2006). Pure tones are chosen as their lack of additional harmonics or timbral features (Baharloo et al., 1998) ensures that no additional cues are used to help participants identify their pitch. Instrumental timbres are rich in contextual detail, and are often easier to identify (Wilson et al., 2012). These are chosen for ecological validity, as they are more representative of musical sounds heard on a daily basis and thus may be better able to capture the extent of an individual’s ability. Other task characteristics related to both the stimuli and to task administration also vary and contribute to ongoing difficulties characterising the AP phenotype.

Given the high degree of heterogeneity among pitch-naming tasks and thresholds, it is unsurprising that a consensus regarding the AP phenotype has not yet been reached. To further advance AP research, particularly the search for genetic variants and biological mechanisms, a phenotype (or phenotypes) must first be clearly defined and accepted. Use of a consistent definition, task parameters and thresholds across studies ensures that findings are comparable and improves replicability in the field. An important first step in this endeavour is to catalogue the current tasks used to profile AP and examine their effects on phenotype identification. In this systematic review, we therefore aimed to 1) investigate the methods and replicability of pitch-naming tasks for the assessment and classification of individuals with AP; and 2) examine the ways in which variability in methods impacts our understanding of the AP phenotype.

Methods

Eligibility Criteria

We included empirical, peer-reviewed original research in which AP was a primary outcome measure, as determined by its inclusion in the title or abstract, excluding case studies and case series. We excluded theses, abstracts, and conference proceedings, and studies published in languages other than English.

Studies were restricted to those with neurotypical adult participants with normal hearing, excluding populations such as those with synaesthesia or autism spectrum disorder.

We were interested in how studies assessed and classified AP, so focused on studies that used: a) a pitch-naming task; and/or b) self-report to determine AP. Studies using self-report alone were included to assess the extent to which our understanding of the AP phenotype is drawn from research that does not include an objective measure of pitch-naming. Studies that developed novel AP measures were included if they also screened their participants with a pitch-naming task, with only the pitch-naming task included in this review. Pitch-naming tasks were limited to those that used conventional Western tuning, excluding those that incorporated stimuli mistuned from the 12 standard chroma, or those that had fundamental frequencies removed. Studies that attempted to either teach AP to novices or to pharmacologically alter pitch perception were excluded, as were studies that screened for AP to exclude AP possessors from their sample. Studies that investigated latent/implicit pitch memory were not considered to be studies of AP as commonly defined.

Search Strategy

We searched the following databases using the search terms “absolute pitch” OR “perfect pitch” on 31^st^ October 2019, restricting results to those published since 1992 to span a 30-year period including: Scopus, PsycInfo, ProQuest Music Periodicals Database, Music Index and JStor (search performed 2^nd^ November 2019). This search was repeated on 31^st^ January 2022 and 23^rd^ May 2024 to capture any studies published since the original search.

Data Collection

Study Selection

JB screened the search results for duplicates, and removed irrelevant papers based on title. This author then screened by title and abstract to determine articles to be retrieved for full-text search and evaluated these full-text results based on the inclusion criteria. Determination of whether AP was a primary outcome measure was agreed upon by discussion with all authors.

Data Extraction

JB extracted data from the selected studies using a template agreed upon by all authors, as shown in Table 1. Data were extracted from information available in the published paper, and were augmented by raw data or supplementary materials where these were available on the relevant journal website. Where studies reported that their methods were available in a previously published paper, these details were extracted and included in the data for the citing study.

Table 1

*Details Extracted from Selected Studies*

| **Definition** | Definition of AP (usually found in the Introduction) |
| --- | --- |
| **Participants** | Total number of participants |
|  | Number of participants in AP/non-AP groups |
|  | Whether non-AP participants were referred to as musicians/musically trained or were non-musicians |
| **Methods** | Method by which AP was determined (pitch-naming task or self-report) |
|  | *Task parameters* |
|  | Whether the pitch-naming task was based upon a previously published task (as determined by direct citation in the Methods section) |
|  | *Stimulus information* |
|  | **Pitch range:** Range of pitches used as stimuli for a given task |
|  | **Timbre:** Timbre(s) in which stimuli were presented |
|  | **Number of trials** |
|  | **Stimulus length (ms)** |
|  | **Response window (ms):** the amount of time in which participants could respond to a trial |
|  | **Response method:** modality by which participants responded to the stimuli (e.g., written, verbal) |
|  | **Inter-trial distracter stimuli:** presence of an additional auditory stimulus between trials |
| **Phenotypic Index** | *Scoring* |
|  | Scoring method (e.g., whether credit assigned for semitone errors) |
|  | Accuracy thresholds for group membership |
|  | Mean performance on pitch-naming task with 95% confidence intervals for participant groups |

*Note.* Where a task was replicated from a previously published task, details from the cited task were used to supplement stimulus information where necessary

Search Results

Searches run on 31st October and 2nd November 2019 yielded 3704 records, 2861 of which remained after removal of duplicates, as shown in Figure 1(A). These records were then screened for relevance, and 266 were retained for full-text retrieval. From these, 128 were removed for not meeting inclusion criteria. During data extraction, a further eight studies were excluded for the same reason (see Table 2 for details).

Additional searches were performed on 31st January 2022 and 23^rd^ May 2024 to capture records published since the initial search (see Supplementary Figure 1). A further 27 articles were identified, resulting in a final total of 157 articles included in the present review. From these, 160 unique studies were identified as three papers included multiple studies with different participants. All included studies are highlighted in the References section, and raw data is included as a supplementary file.

Figure 1

*Search Strategy for 2019 Search*

*Note.* Further details for removal of studies not meeting inclusion criteria can be found in Table 2.

Table 2

*Reasons for Study Exclusion*

|  | Reason for exclusion | N |
| --- | --- | --- |
| Removed following full-text retrieval (Oct 2019 search) | Not original research   - Commentary/errata - Review | 15  26 |
|  | AP not primary outcome measure | 4 |
|  | Paper not in English | 24 |
|  | Non-adult sample   - Children - Computational model (no participants) | 5  1 |
|  | Not neurotypical/normal hearing | 1 |
|  | AP training | 6 |
|  | Pharmacological studies | 7 |
|  | AP possessors screened out | 4 |
|  | Case studies | 1 |
|  | Conference papers | 4 |
|  | Latent AP | 17 |
|  | Not relevant from full text | 13 |
| Removed during data extraction | AP not primary outcome measure | 3 |
|  | Non-pitch-naming task used | 5 |
| Removed following full-text retrieval (Jan 2022 search) | Not original research   - Registered report protocols | 1 |
|  | AP not primary outcome measure | 3 |
|  | AP participants excluded | 1 |
|  | Not relevant from full text | 15 |
| Removed following full-text retrieval (May 2024 search) | AP not primary outcome measure  Non-pitch-naming task used | 1  1 |
|  | AP participants excluded | 5 |
|  | Not relevant from full text | 6 |

Data Analysis

Data were presented graphically to show the variety in approaches to pitch-naming tasks across the literature. Where necessary, means and 95% confidence intervals were calculated from raw data or other reported summary statistics.

To map the relationships between tasks used by different research groups, we developed pitch-naming publication ‘trees’ that identify ‘source tasks’ used in early studies and show the flow of publications that stem from each source task as cited in the methods of each study.

To investigate how differences in task parameters impacted the AP phenotype, the performance means from the studies’ AP groups were compared across each parameter, using correlations or two-tailed *t*-tests as appropriate. Only those studies using the same scoring practices were included in these tests to ensure consistency of comparisons.

All analyses were performed in RStudio (version 2023.06.2+561), using packages *tidyverse,* version 2.0.0 (Wickham et al., 2019), *psych,* version 2.3.6 (Revelle, 2024), *scales,* version 1.2.1, (Wickham et al., 2022), *forestplot,* version 3.1.3, (Gordon & Lumley, 2024), and *lattice*, version 0.21-8 (Sarkar, 2008).

Results

Definition of AP

The definition of AP was extracted from each study as a direct quote (see Supplementary Table 1). Most studies agreed that AP refers to the ability to identify notes without a reference tone, with some also including the ability to produce notes without reference. In total, 150 of the 151 studies (99%) specifying a definition agreed on this, with a single study providing a definition referencing neither identification nor production, but instead highlighting long-term pitch memory (Wayman et al., 1992). The remaining six studies did not provide a definition for AP.

Participants

Details of study participants are shown in Table 3. Across 160 studies, there was a total of 23,221 participants. This figure, however, does not account for potential participant overlap among studies, so the number of unique participants is likely to be somewhat smaller but difficult to estimate as not all studies reported this. Of the total participants, 6,493 were classified as having AP. This figure is an estimate, as some studies reported the percentage of the total sample to have AP rather than raw *N*. Participants classed as neither AP nor non-AP were classified as intermediate pitch-namers (*n* = 1,133), non-musicians (*n* = 964), or were in studies that did not group participants into AP categories (*n* = 3,545). These do not sum to the total of 23,221 as some participants were initially assessed but not included in a study’s final group classification. Both AP and non-AP groups were generally quite small in individual studies (median AP group *n* = 16). Most studies including an AP group used participants with musical experience as a comparison group (137/145 studies, 94%), with 21% (31/145 studies) also including a group without musical experience.

Table 3

*Studies and Participants*

| Studies | *N* | Range | *Mean* (*SD*) | Median |
| --- | --- | --- | --- | --- |
| Number of studies | 160 |  |  |  |
| Total participants across studies | 23,221 | 5-2,707 | 145.1 (365.4) | 41 |
| Total AP participants across studies | 6,520 | 0-1508 | 42.9 (147.7) | 16 |
| Total non-AP participants across studies | 10,222 | 2-2458 | 75.2 (260.0) | 18 |
| Studies classifying participants by pitch-naming performance | 152 (89%) |  | | |
| Studies using participants with musical experience as a comparison group | 137/145 (93%) |  | | |

Pitch-Naming Tasks

Most studies (150/160, 94%) used a pitch-naming task to classify participants as belonging to an AP or non-AP group based on their performance. It should be noted that tasks did not provide feedback to participants throughout pitch-naming procedures. Five studies (5/160, 3%) used a pitch-naming task but considered AP to be a continuous ability, so participants were not divided into AP/non-AP groups. Only three studies (3/160, 2%) relied on self-report alone for AP group assignment, and two studies (2/160, 1%) did not describe how they determined group membership.

Three studies (Keenan et al., 2001; Ngan et al., 2023; Schulze et al., 2013) used two separate pitch-naming tasks to assign AP group membership. As these were part of the group determination, rather than novel tasks designed to follow initial AP classification, both tasks are considered here. Both tasks used sine tones of equal duration, but differed in the pitch range of stimuli and number of trials.

Pitch-Naming Publication Trees

In total, 157 tasks were used to measure pitch-naming performance. Of these, 95 (61%) were either direct replications or adaptations of previously published tasks. Over a third of the pitch-naming tasks described in the literature were therefore either novel or did not explicitly cite a basis for their pitch-naming methods. Pitch-naming publication trees showing the relationships between tasks are shown in Figure 2 and Supplementary Figure 2.

The first of these set of publication trees (Figure 2(A)) indicates that while six influential papers have formed the basis of many pitch-naming tasks, there is limited replication across research groups. However, these tasks are used in subsequent publications by the same research groups. In particular Figure 2(A) shows that there are no links between the six publication trees, rather only links within each tree. A further issue is the degree of modification to the source task in subsequent studies, which limits the extent to which a source task can be said to be ‘replicated’. Modifications may be minor, such as Athos et al. (2007) shifting the Baharloo et al. (1998) paradigm to be delivered online, or adjustments that change the number and range of trials as well as stimulus timbre (e.g., Weisman et al., 2012). Some modifications are subsequently employed across multiple papers (e.g., a single adaptation is used across all adapted tasks derived from the shared Oechslin, Imfeld et al., 2010/Oechslin, Meyer et al., 2010 paradigm). Figure 2(B) shows tasks derived from reviews rather than individual studies. These reviews synthesise an understanding of methodological choices and inform how subsequent researchers choose to construct their own distinct tasks. Further tasks are included in Supplementary Figure 1, each of which have been used or modified in a limited number of subsequent studies.

Figure 2

*Publication Trees Showing Relationships Among Tasks and their Replication*

*Note.* Studies are linked by arrows, with the arrowhead pointing towards the study that cites the previous study’s task. Dotted lines are for readability and are used the same way as solid lines. Panel (A) shows tasks used in multiple subsequent studies. Tasks that are direct replications of their parent task are in plain text, while those that are adaptations are in grey. Panel (B) shows tasks derived from reviews. No replication/adaptation distinction is made here as the source papers do not include specific tasks.

Characterising the AP Phenotype

Scoring

The most common method of scoring pitch-naming tasks was to count the number of correct responses. Other scoring methods, usually used in conjunction with the total accuracy score, included mean absolute deviation from the target tone (e.g., Bermudez & Zatorre, 2009; Dohn et al., 2014), internal consistency of responses (rather than objective correctness, used in Burns & Campbell, 1994), and tallying octave errors (e.g., Bahr et al., 2005).

In considering total accuracy scores, some were raw scores consisting of the sum of correct responses, while others also assigned partial or full credit for semitone errors. Out of 151/157 tasks reporting total scores, 41/151 (27%) assigned semitone credit when determining AP group membership, with the remaining 110/151 (73%) using raw scores. Some studies utilised both raw and semitone-credit scoring systems (e.g., Li, 2021) but used raw scores alone to classify AP. Credit for semitone errors included 0.25 points (*n* = 1), 0.5 points (*n* = 9), 0.75 points (*n* = 9), one full point (*n* = 13), or varying credit depending on participant age (*n* = 5).

Thirty-two tasks (32/151, 21%) required participants to identify the octave alongside the chroma label, but octave errors were usually considered a separate metric rather than contributing to the raw accuracy score.

Thresholds

Studies that specified accuracy thresholds to determine AP group membership are shown in Figure 3. The strictest threshold for classifying AP was 100% pitch-naming accuracy (Matsuda et al., 2013), while the least stringent raw score threshold was 20% (Maeshima et al., 2018). Where semitone error credit was applied, thresholds were less conservative than those that only considered raw scores. For raw scores, the mean AP threshold was 77% (*SD* = 20, median = 85%), while it was 71% (*SD* = 16, median 68%) for studies including semitone error credit.

While most studies included both AP and non-AP performance groups, only 32/95 (34%) that included an AP classification threshold also specified a threshold for non-AP performance. Eighteen studies (19%) of the 95 reporting AP thresholds also considered intermediate levels of performance (e.g., QAP). This was usually classed as a single intermediate group, although was sometimes further broken down into 10% performance bands (Miyazaki et al., 2012; Miyazaki et al., 2018).

Figure 3

*Accuracy Thresholds Used Across Studies*

**

*Note.* Scores classified into AP, non-AP, and intermediate groups are shown by shaded bars. Green bars refer to AP performance, red bars to non-AP performance, and teal/blue/orange refer to intermediate groups. Lighter versions of the colours (e.g., Gruhn et al., 2018) indicate studies for which semitone error credit was applied. Hou et al. (2014, 2021, 2023) include a cross-hatching over the AP group to denote that only white-key notes were used in this task. Diagonal fill indicates that no non-AP groups completed the pitch-naming task in these studies. Asterisks next to study names indicate that additional metrics were used beyond these thresholds to determine group membership. ^a^This paper is represented twice as it contains two separate studies. ^b^*N* includes a non-musician group that did not complete the pitch-naming task.

Mean Performance

While thresholds represent the potential limits of classification of performance on a pitch-naming task, some studies also reported actual participant performance. Figures 4.0 – 7.0 contain forest plots of mean AP and non-AP participant performance with 95% confidence intervals (CI). Separate plots are shown for studies that used raw accuracy scores (Figures 4.0 and 6.0) and those that included semitone error credit (Figures 5.0 and 7.0) given these metrics are not directly comparable.

The mean AP performance across 58 studies was 85.9% (95% CI 83.1 – 88.8%), while performance in the 25 studies assigning semitone error credit was 89.1% (95% CI 86.3 – 91.8%). The mean non-AP performance across 50 studies was 17.0% (95% CI 14.0 – 20.0%) based on raw accuracy scores (where chance performance is 8.3%), and 24.5% (95% CI 19.7 – 29.3%) for the 19 studies assigning semitone error credit.

Figure 4

*Mean Performance of AP Participants in Studies Using Raw Accuracy Scores*

*Note.* Error bars are 95% confidence intervals around the mean (omitted when relevant data were unavailable). The mean is shown by the grey vertical line.

Figure 5

*Mean Performance of AP Participants in Studies Assigning Credit to Semitone Errors*

**

*Note.* Error bars are 95% confidence intervals around the mean (omitted when relevant data were unavailable). The mean is shown by the grey vertical line.

Figure 6

*Mean Performance of Non-AP Participants in Studies Using Raw Accuracy Scores*

**

*Note.* Error bars are 95% confidence intervals around the mean (omitted when relevant data were unavailable). The blue vertical line indicates chance performance (8.3%), and the mean is shown by the grey vertical line.

Figure 7

*Mean Performance of Non-AP Participants in Studies Assigning Credit to Semitone Errors*

**

*Note.* Error bars are 95% confidence intervals around the mean (omitted when relevant data were unavailable). As chance performance varies according to the amount of credit assigned to semitone errors, a chance line is not included. The mean is shown by the grey vertical line.

The Influence of Task Parameters on the Expression of the AP Phenotype

Pitch Range

The majority of tasks specified the stimulus pitch range (139/157 tasks, 89%). As shown in Figure 8(A), almost all tasks reporting a range included the central octave (C4 – B4), with the exception of six studies: one that used just one trial in its pitch-naming task (Van Hedger et al., 2016), another that used 10 specific chroma between G#1 and G6 (Di Giuseppe Germano et al., 2021), one which used two tasks – the first of which used the range C5 – B5 (Ngan et al., 2023), and three that used only the white notes from the central octave (Hou et al., 2014, 2021, 2023). Range varied from a single octave to over eight octaves, exceeding the range of a piano, with the majority of studies using a range that spanned three octaves (see Figure 8(A)). A Pearson correlation analysis of the pitch range and mean performance of the AP group in each study using raw accuracy scores (*n* = 50) showed that performance did not differ according to the range used (*r*(48) = -.14, *p* = .320, 95% CI [-.41, .14], see Figure 8(B) for all studies regardless of scoring method).

Figure 8

*Pitch Range of Pitch-Naming Task Stimuli*

*Note.* Panel (A) shows the pitch range as reported for 139/157 tasks. Each blue line represents a single task, with endpoints representing the upper and lower limits of each task’s specified range. Middle C (C4) is indicated with a red vertical line, while the range of a piano is shown by green vertical lines. Panel (B) shows the correlation between mean pitch-naming performance and task stimulus range for all tasks regardless of scoring method, *n* = 77.

Timbre

The timbre used for pitch-naming stimuli was a highly reported task parameter, with 151/157 tasks (96%) reporting this information. As shown in Figure 9(A) the two most common timbres were sine and piano tones, used as stimuli in 125/151 (83%) tasks. Other timbres used were synthesized complex tones and voice. The remaining 19 studies (13%) used multiple timbres within their tasks. Of these, 16 included piano tones, and 11 included sine tones. Other timbres included: triangle tones (3) harpsichord (1), guitar (2), violin (6), organ (2), unspecified woodwind (2), unspecified brass (2), voice (5), unspecified string (1), cello (1), flute (2), clarinet (1), bassoon (1), trumpet (1), trombone (1), French horn (1), tuba (1), “random” (1), synthesised complex (1), viola (3), synthesised voice (2), smooth tones (1), and participant’s own instrument (1). Among studies reporting mean performance scores for raw accuracy, those using piano tones only (*n* = 43) reported higher scores (*M* = 93.0, *SD* = 6.1) than those using sine tones only (*n* = 39, *M* = 81.4, *SD* = 9.4; *t*(43.97) = 5.06, *p* < .001, 95% CI [6.97, 16.21]). Figure 9(B) shows the mean performance of AP groups across all timbres, without accounting for different scoring methods.

Figure 9

*Timbres Used in the Pitch-Naming Tasks*

**

*Note.* Panel (A) shows the proportion of tasks using different timbres, while (B) shows the mean pitch-naming performance of AP groups for these different timbres. Error bars are 95% confidence intervals around the mean.

Number of Trials

Stimulus presentation and response characteristics of the pitch-naming tasks are shown in Table 4, including details of the number of task trials. Across tasks for which the number of trials was reported (152/157, 96.8%), the most common number was 108 trials, as a result of multiple published studies using the 108-trial paradigm developed by Oechslin, Imfeld et al. (2010; Oechslin, Meyer et al., 2010; see Figure 2(A)). This paradigm presents each chroma nine times. Importantly, the majority of tasks included sufficient trials for each chroma to be presented more than once (≥ 24 trials), with a median of 60 trials.

The relationship between the number of trials and pitch-naming performance is shown in Figure 10 incorporating all studies reporting the mean for their AP group regardless of scoring method. The figure shows a strong negative correlation with performance accuracy sharply decreasing with an increasing number of trials (*r*(82) = -.47, *p* < .001, 95% CI [-.62, -.28]). This remained significant even when excluding the outlier in the bottom right of the figure (*r*(81) = -.26, *p* < .02, 95% CI [-.45, -.05]; Bahr et al., 2005). When considering those studies reporting mean raw accuracy scores alongside the number of trials (*n* = 57), the negative correlation between the number of trials and task performance remained large (*r*(55) = -.64, *p* < .001, 95% CI [-.77, -.46]).

Table 4

*Stimulus Presentation and Response Characteristics of the Pitch-Naming Tasks*

| Characteristic | *N* tasks reporting (%) | Mean (*SD*) | Median | Mode (frequency) | Range |
| --- | --- | --- | --- | --- | --- |
| Number of trials | 152/157 (96.8%) | 76.0 (85.4) | 60 | 108 (31) | 1-960 |
| Stimulus duration | 119/157 (75.8%) | 847.5 ms (449.1) | 1000 ms | 1000 ms (54) | 100-3000 ms |
| Response window | 100/157 (63.7%) | 4778.4 ms (3451.4) | 4000 ms | 4000 ms (23) | 1000 ms - self-paced |
| Distracter stimuli | 32/157 (20.3%) |  |  | Brown noise (18) |  |

Figure 10

*Mean Pitch-Naming Performance of AP Groups for Tasks with Varying Numbers of Trials*

Stimulus Duration

Summary statistics for stimulus duration indicate that most studies used either 500 or 1,000 ms tones (see Table 4), although this characteristic was less consistently reported than other parameters (119/157 tasks, 75.8%). A Pearson correlation analysis indicated that pitch-naming task accuracy for studies using raw scores (*n* = 46) was not associated with stimulus duration (*r*(44) = .00, *p* = .974, 95% CI [-.29, .29]; see Figure 11 for all tasks regardless of scoring method, *n* = 61).

Figure 11

*Mean Pitch-Naming Performance of AP Groups for Tasks with Varying Stimulus Duration*

Response Window

While the majority of tasks (100/157, 63.7%) included information on response windows (see Table 4), it was somewhat difficult to quantify the typical period allowed for participant responses. This was because it was often unclear whether the response window was inclusive of the duration of the presented stimulus, with inconsistent reporting between studies purportedly using the same task. Based on studies using raw scores in which this information was clear (*n* = 40), a Pearson correlation analysis showed that pitch-naming accuracy did not differ according to the length of the permitted response window (*r*(38) = -.26, *p* = .100, 95% CI [-.53, .05]). Figure 12 shows all tasks regardless of scoring method (*n* = 59), excluding the three tasks that reported responses as self-paced.

Figure 12

*Mean Pitch-Naming Performance of AP Groups for Tasks with Varying Response Window*

Response Method

Details of how participants were asked to name pitch stimuli were provided for 119/157 (76%) tasks. The most common method was for the participant to write the chroma name down (52 studies). Other methods included: (i) indicating the correct chroma on a piano keyboard (either a physical [muted] or visual representation; *n* = 19); (ii) selecting an onscreen chroma label (*n* = 23); (iii) pressing a labelled computer key or response button (*n* = 13); (iv) responding verbally (*n* = 9); or (v) writing the correct note on a musical staff (*n* = 3). One study presented its pitch-naming task twice to participants, one using an onscreen label to record responses and the other using an onscreen piano keyboard, with no performance difference found between these response methods (Brancucci, Dipinto, et al., 2009). Pitch-naming accuracy performance for the various response methods is shown in Figure 13. It indicates that variability is lowest when the response is a labelled button, although this is somewhat misleading as two of the nine tasks using this method and reporting mean AP group performance used the same sample (Hsieh & Saberi, 2008a; Hsieh & Saberi, 2009), while another two were separate tasks completed by the same participants in the same study (Ngan et al., 2023). Use of a piano key response produced the least accurate and most variable responding, though these studies also used low thresholds for AP group membership (40 – 79%), which may explain the relatively low performance here. Overall, AP group performance was variable across all response methods, and as the number of tasks per response method is limited (from *n* = 2 to *n* = 26 per method that report mean AP group performance), it is difficult to draw firm conclusions regarding the relationship between response type and pitch-naming accuracy.

Figure 13

*Mean Pitch-Naming Performance of AP Groups Across Response Methods*

*Note.* All tasks reporting the mean for their AP group are included in this figure regardless of scoring method (*n* = 66). Error bars are 95% confidence intervals around the mean.

Inter-Trial Distracter Stimuli

Most studies did not include additional auditory stimuli between response trials (see Table 4). Of the 32/157 (20%) that did, 18 used brown noise, seven used white noise, three used distorted tones, and four used a rapid sequence of notes or glissando. As shown in Figure 14, pitch-naming accuracy was lower in studies using distracter stimuli, with a comparison between studies reporting raw accuracy scores (*n* = 59) indicating a significant difference (*M_no sound_* = 88.79, *SD* = 9.88, *M_sound_* = 82.06, *SD* = 11.00; *t*(45.92) = 2.40, *p* = .021, 95% CI [1.08, 12.37]).

Figure 14

*Mean Pitch-Naming Performance of AP Groups According to the Presence of a Distracter Sound*

*Note.* All studies reporting the mean for their AP group are included in this figure regardless of scoring method. Error bars are 95% confidence intervals around the mean.

Discussion

In investigating the methods of pitch-naming tasks, we found that researchers near-universally agree on the conceptual definition of AP. The ability of AP possessors to identify and label isolated musical tones is the foundation of our understanding of this phenotype. The broad definitional uniformity across studies of AP is, in a sense, a validity check of the selection criteria for this review. One would expect a degree of consensus across studies for which AP is a primary outcome measure, and which largely rely on pitch-naming tasks as the method of assessment. There is a lack of coherence, however, in how this core feature is best measured by pitch-naming tasks. With close to 40% of studies using unique pitch-naming paradigms, there is a sense that AP studies are constantly ‘reinventing the wheel’.

The high degree of heterogeneity in both pitch-naming methods and the accuracy of AP group performance reflects a relative lack of maturity in the field of AP research. Linden and Hönekopp (2021) argue that high heterogeneity indicates a mismatch between data and concept, and that reduction of this disparity is necessary for fields of research to progress. Although we did not employ formal heterogeneity measures for effect size (e.g., *I^2^*, Borenstein et al., 2021), the data in this review nevertheless point to a heterogeneous understanding of AP in how we translate a broad conceptual understanding to a specific, measurable phenotypic index. To move AP research to a more mature field of study, we must explore the sources of this heterogeneity and address them from both a methodological and theoretical perspective. Our review aims to primarily target the methodological aspect of this challenge, though our recommendations below are theoretically informed.

A major contribution of this review is to demonstrate the ways in which variability in methodological choices for specific task parameters impacts expression of the AP phenotype. Perhaps the most striking example of this is the choice of accuracy thresholds for AP classification. Figure 4 presents a clear picture of the heterogeneity in the levels of pitch-naming performance considered to characterise AP. We acknowledge that this is a somewhat simplified view, as some studies use multiple metrics to classify AP rather than thresholds alone (e.g., response time in Van Hedger et al., 2018; mean absolute deviation alongside accuracy scores in Chavarria-Soley, 2016). Even taking this into account, however, it is notable that the AP phenotype is often characterised by pitch-naming performance that overlaps with other partial phenotypes or even non-AP performance, especially when scoring differences such as semitone errors are considered. These scoring differences make it difficult to directly compare studies across various scoring metrics. If researchers choose to assign credit to semitone errors, reporting would be improved by including both raw and error-corrected scores. This would ensure that studies can be more easily compared, rather than dividing them into raw and error-corrected categories as we have needed to do here.

Exploring participant performance yields information beyond the potential limits defined by thresholds. Analysis of mean performance shows that pitch-naming ability is a dimensional trait, with scores lying along a spectrum from chance to ceiling, regardless of participant classification into AP and non-AP groups. To score above chance on a pitch-naming task, participants must have some degree of pitch-naming ability. However, the forest plots and threshold plot show that above-chance participants are sometimes included in the ‘non-AP group’, which reduces the discriminatory power of studies to find differences between AP and non-AP participants, and thus accurately characterise the AP phenotype. Furthermore, the use of thresholds (particularly *a priori* thresholds) assumes that AP can meaningfully be divided into discrete categories, perpetuating dichotomous AP/non-AP classification in a somewhat circular manner between measurement and conceptualisation and reinforcing existing definitions of AP phenotypes. This review acts as an essential step in the development of a taxonomy of AP phenotypes, stepping away from the problem of circularity.

A further complication is that non-AP scores have not been consistently reported, so the performance of the comparison group cannot always be gauged from the published work. Ideally, thresholds should be set at or around chance performance (8.3%) to ensure that all degrees of pitch-naming ability are being captured, including intermediate forms, such as QAP. Consideration of participants across the full range of ability would then allow characterisation of different pitch-naming phenotypes that differ in their pitch-naming accuracy, as well as the cognitive strategies used, and the extent of specificity to contextual cues such as timbre. Data-driven techniques such as taxometric analysis (Ruscio et al., 2006) should be used to assess the extent to which these phenotypes are discrete by employing multiple AP tasks, rather than relying on a threshold from a single pitch naming task that is necessarily arbitrary. This will move the field towards a more robust method of phenotyping AP.

Each task parameter investigated in this review varied considerably across studies, including in how consistently it was reported. No single parameter was described across all 157 tasks. The number of trials was reported most reliably, followed by stimulus timbre, pitch range, participant response method, stimulus duration, and response window. Omitting key details from published methods reduces our ability to assess the replicability of findings and contributes to the continued development of novel pitch-naming tasks, as evident from the pitch-naming publication trees.

Due to the significant variability among pitch-naming task methods, it is difficult to assess the effects of specific task parameters on expression of the AP phenotype. However, where possible we examined whether systematic variation in a specific task parameter was associated with varying expression of the AP phenotype. From this, we have derived some initial recommendations for future studies to promote greater homogeneity in measuring the AP phenotype by endorsing key characteristics that should be captured and reported by a gold-standard task.

Recommendations for Pitch Range and Task Trials

The pitch range of the stimuli and the number of trials over which they are presented are a matter of content validity – that is, whether pitch-naming tasks adequately canvas the range of behaviours AP possessors are expected to exhibit. The conceptual definition of AP does not place an upper or lower limit on the number or pitch range of chroma that AP possessors are expected to be able to identify. It is assumed that AP possessors can effortlessly identify all 12 chroma (although see Miyazaki, 1988 for a discussion regarding preferred chroma even among highly accurate AP participants). As such, a bare minimum of 12 trials, each representing a different chroma, would be a basic starting point. However, a single trial per chroma is unlikely to be sufficient to fully capture participant ability. In particular, a limited number of trials may mask the variability of intermediate-level performance, and thus we caution against relying on too few trials per participant. The reduction in pitch-naming performance as trial numbers increase is somewhat more challenging to interpret. This effect is largely driven by the high degree of variability across studies using 108 trials. This number of trials is shared across multiple paradigms, including those derived from Bermudez and Zatorre (2009) and Oechslin, Imfeld et al. (2010)/Oechslin, Meyer et al. (2010) as shown in Figure 2(A). This effect therefore is likely to reflect the popularity of tasks using this number of trials (allowing each chroma to be presented nine times) across different accuracy thresholds (from 40%, Kamiyama et al., 2010; to 90%, Coll et al., 2019), rather than an implication that actual participant performance decreases as trial numbers increase. This could be further investigated by checking performance in earlier versus later trials in lengthier paradigms.

The pitch range that trials should cover is similarly unclear, with no significant impact of pitch range on mean task performance shown across studies. Most studies reporting range included, at minimum, the central octave on the piano (C4 – B4). Previous research has indicated that pitch-naming accuracy tends to decline at the extremes of the pitch range (Miyazaki, 1989; Takeuchi & Hulse, 1993; West Marvin et al., 2020), but there is no clear expectation of the range in which good performance should occur in AP possessors. Rakowski and Rogowski (2011) suggest a five-octave range based on an investigation using sine tones, in which participant accuracy declined beyond this range. They did, however, note heterogeneity in performance among their small sample, so this is not a universal feature of AP possessors. It is therefore yet to be established whether a single-octave range is too narrow to appropriately gauge AP, or if an eight-octave range unnecessarily contributes to an excessive number of trials. Stimulus range may be pertinent to distinguishing between phenotypes, as per the suggestion of ‘universal’ versus ‘limited’ (range) AP (Bachem, 1937). As such, tasks aiming to make this distinction should include stimuli across a wide pitch range, and include range-related accuracy analysis rather than just raw task-wide performance. At this point, however, the contributions of contextual factors such as range to AP phenotypes need to be further elucidated, so a separate pitch-naming task that measures the limits of range may be appropriate alongside a gold-standard pitch-naming task that is comparable across studies. Based on the most common range and trial numbers among studies in this review, we recommend a pitch range that captures three octaves and uses at least 5 trials per chroma. This balances: i) the need to canvas a sufficient range that most AP possessors can be expected to identify; ii) multiple trials per chroma; and iii) a sufficiently short task duration to enable additional tasks to be administered as needed to distinguish specific phenotypes.

Recommendations for Stimulus Timbre

Stimulus timbre, as shown in this review, is largely divided between piano tones and sine tones, with scores on tasks using a piano timbre exceeding those using sine tones. This highlights a divide in the understanding of how AP is conceptualised – prioritising either stimulus ‘purity’ or ecological validity. The ecological validity argument emphasises the importance of context for the AP phenotype, not only in terms of timbral cues, but also the context in which the long-term pitch memory was originally encoded. There is strong evidence supporting a critical or sensitive period for AP acquisition, including early practice on the piano (Bairnsfather, Osborne, et al., 2022; Deutsch et al., 2006; Levitin & Zatorre, 2003; Russo et al., 2003; Vanzella & Schellenberg, 2010; Wilson et al., 2012). This indicates that early environmental factors are influential in shaping the expression of the AP phenotype, suggesting AP is a contextually-learned behavioural skill rather than a purely psychophysical phenomenon. Neural encoding of the pitch template may be contextually specific, as is seen in increased cortical representations among musicians for piano, but not sine tones (Pantev et al., 1998). The idea of ‘universal’ and ‘limited’ AP phenotypes is again relevant here (Bachem, 1937). Some AP phenotypes may be more contextually bound than others, with individuals able to identify chroma across a limited or broad range of timbres. Tests of AP should, therefore, aim to capture this variability, with piano tones or other personally-tailored timbres more able to do this than context-devoid sine tones. Supporting this, studies including both piano and sine tones generally show a drop in sine tone performance accuracy (Athos et al., 2007; Hsieh & Saberi, 2008b; Lee et al., 2011; Miyazaki, 1989), reflecting that use of sine tones alone risks failing to fully capture the AP phenotype. We recommend that a gold-standard AP task should use the piano timbre as a contextually relevant, ‘neutral’ stimulus. Additional timbres, particularly sine tones, can be utilised in subsequent, specific tasks, depending on the phenotype targeted in individual studies. This would allow the potential limits of AP phenotypes to be tested.

Recommendations for Stimulus Duration, Participant Response Methods, and Distracters

Other task characteristics, such as stimulus duration and response window, also vary between studies, and are less frequently reported than timbre, range, and the number of trials. In this review, these parameters of pitch-naming tasks do not significantly contribute to differences in phenotypic expression, although noting that response window was not always reported clearly. Most commonly, it is not specified whether the permitted response window is inclusive of the time to deliver the stimulus (e.g., Dohn et al., 2012 vs Dohn et al., 2015). Inclusion of a schematic clearly showing task presentation and timing, as is common in cognitive psychology paradigms, would reduce this ambiguity. As duration and response window do not appear to greatly influence the AP phenotype, we recommend using the most commonly reported methods to maximise comparability among studies – 1,000 ms stimulus length, with 4,000 ms response window excluding the stimulus duration.

The review also shows that response methods vary widely among tasks, with each associated with different levels of pitch-naming accuracy. The reason for these discrepancies is likely multifactorial and associated with other task parameters alongside the response method used, such as the allowed response window (e.g., writing the response on a musical staff requires i) knowledge of music notation, and ii) more time than pressing a response key). Our recommendation is to avoid response methods that disadvantage some participants, such as piano keys that may be less familiar to non-pianists, or staff notation that requires participants to be able to read music. Response methods such as key/button press or clicking an onscreen button may be particularly useful, as they facilitate the precise capture of response time.

As distracter stimuli between trials are associated with lower participant accuracy, this suggests that they are fulfilling their purpose of preventing relative pitch strategies being used across trials. It would be appropriate, therefore, to recommend their use in a gold-standard pitch-naming task.

Limitations of this Review

While this review aimed to canvas a large part of the AP literature, it is by no means exhaustive. Further heterogeneity is apparent in studies beyond the scope of the current review, such as those in which AP was not the primary focus of investigation (e.g., Acevedo et al., 2014; Matsunaga & Abe, 2005; Pfordresher & Kobrina, 2017). Such studies are more likely to rely on self-report of AP possession rather than objectively measuring pitch-naming performance. The validity of self-report as a measure of AP ability is a useful question for further research, though first requires consensus regarding the phenotype that self-reported AP possessors claim to have. Attempts have also been made to measure AP beyond pitch-naming tasks, such as pitch production (Heald et al., 2014), a go/no-go discrimination task (Weisman et al., 2012), Stroop-like tasks (Leipold, Greber, et al., 2019; Schulze et al., 2013), and pitch-naming tasks that test the limits of AP by omitting frequencies or mistuning stimuli (Gruhn et al., 2018; Hsieh & Saberi, 2009; Rogowski & Rakowski, 2010). These tasks may be particularly useful in validating preliminary phenotypes characterised using data-driven analysis of pitch-naming performance, and potentially expanding the number of recognised phenotypes or the features of a given phenotype. Such tasks can also be used to explore AP predisposition among individuals without musical training.

Conclusions

As research into the genetic underpinnings of behavioural traits increases, the necessity for well-described phenotypes is of renewed interest. Indeed, among the aims of the recently founded Musicality Genomics Consortium (<https://www.mcg.uva.nl/musicgens/>) is the development of “scalable and robust phenotypes” and the harmonisation of “existing measures of musicality phenotypes” (<https://www.mcg.uva.nl/musicgens/mission.html>). This review is therefore timely and shows how far we still have to go in developing phenotypes for AP.

Overall, this review has shown that while there is strong consensus regarding the conceptual definition of AP in terms of its core features, this does not extend to the methods used to measure pitch-naming ability. The concept is extremely broad and captures many aspects of behaviour, lending itself to varied interpretations when attempting to define AP phenotypes and thus, design tasks to capture them. This lack of precision has led researchers to develop disparate metrics and adopt arbitrary thresholds for AP possession, and there remains no gold-standard pitch-naming task with clearly defined parameters and scoring methods. This has resulted in a highly variable body of literature, with a multitude of pitch-naming tasks differing across all parameters. This, combined with differences in scoring and thresholds to qualify a participant as possessing AP, has resulted in substantial heterogeneity in what is considered to be the AP phenotype. Without a well-described and accepted phenotype, behavioural findings may not be comparable or replicable.

The recommendations we have provided are an important initial step in addressing this. In place of a single task that can capture every phenotypic difference, we advocate for a task that is used across the literature and facilitates replication across studies. Specific phenotypic distinctions can be teased out with subsequent tasks that explore facets such as timbral and range differences. A gold-standard AP task should include multiple (we suggest at least five) trials per chroma to appropriately capture performance variability, spanning three octaves to maximise comparability with existing measures. Stimuli should be piano tones, again to maximise replicability, and to ensure that the timbre is contextually relevant across participants. Additional timbres can be considered in further tasks depending on the phenotypes relevant to the research question. Stimulus length should be 1,000 ms, with a 4,000 ms response window excluding the stimulus duration. While a variety of response methods is likely to be appropriate depending on the research setting (e.g., lab-based versus online task delivery), eliminating the need for participants to be familiar with piano keyboards or music notation will allow the task to be used across a wider range of participants. We also recommend that distracter stimuli are used between trials to ensure that participant performance is not impacted by previously presented material.

Precise phenotyping is vital for genetic research, to ensure that shared genetic variants can be confidently linked to AP rather than to broader or related traits. Moreover, the variability in pitch-naming performance suggests that there may be multiple phenotypes relating to the spectrum of pitch-naming ability. Given the degree of heterogeneity in the current AP literature, an important next step is to characterise intermediate pitch-naming ability. This will help to clarify its relationship to AP and establish assist in determining accuracy thresholds for AP classification. Combined with the findings of previous work exploring different types of AP, more precise phenotypes could then be characterised, forming an empirical basis on which to continue the search for genetic variants for AP.

**Declarations**

**Funding**

The authors did not receive support from ay organisation for the submitted work.

**Conflicts of Interest**

The authors declare no conflicts of interest pertaining to this manuscript.

**Ethics Approval**

As a review of published data, no ethics approval was required.

**Consent to Participate**

Not applicable

**Consent for Publication**

Not applicable

**Data & Code Availability**

The review was not preregistered. Data extracted from studies are available as a supplementary file, and R scripts are available from authors on request.

References

References marked with an asterisk are included in the systematic review.

Abdellaoui, A., & Verweij, K. J. H. (2021). Dissecting polygenic signals from genome-wide association studies on human behaviour. *Nature Human Behaviour*, *5*, 686-694. https://doi.org/10.1038/s41562-021-01110-y

Acevedo, S., Temperley, D., & Pfordresher, P. Q. (2014). Effects of metrical encoding on melody recognition. *Music Perception*, *31*, 372-386. https://doi.org/10.1525/mp.2014.31.4.372

*Akiva-Kabiri, L., & Henik, A. (2012). A unique asymmetrical stroop effect in absolute pitch possessors. *Experimental Psychology*, *59*, 272-278. https://doi.org/10.1027/1618-3169/a000153

*Aruffo, C., Goldstone, R. L., & Earn, D. J. D. (2014). Absolute judgment of musical interval width. *Music Perception*, *32*, 186-200. https://doi.org/10.1525/MP.2014.32.2.186

*Athos, E. A., Levinson, B., Kistler, A., Zemansky, J., Bostrom, A., Freimer, N., & Gitschier, J. (2007). Dichotomy and perceptual distortions in absolute pitch ability. *Proceedings of the National Academy of Sciences of the United States of America*, *104*, 14795-14800. https://doi.org/10.1073/pnas.0703868104

Bachem, A. (1937). Various types of absolute pitch. *Journal of the Acoustical Society of America*, *9*, 146-151. https://doi.org/10.1121/1.1915919

Bachem, A. (1940). The genesis of absolute pitch. *Journal of the Acoustical Society of America*, *11*, 434-439. https://doi.org/10.1121/1.1916056

Bachem, A. (1948). Note on Neu's review of the literature on absolute pitch. *Psychological Bulletin*, *45*, 161-162. https://doi.org/10.1037/h0063411

*Baharloo, S., Johnston, P. A., Service, S. K., Gitschier, J., & Freimer, N. B. (1998). Absolute pitch: An approach for identification of genetic and nongenetic components. *American Journal of Human Genetics*, *62*, 224-231. https://doi.org/10.1086/301704

*Baharloo, S., Service, S. K., Risch, N., Gitschier, J., & Freimer, N. B. (2000). Familial aggregation of absolute pitch. *American Journal of Human Genetics*, *67*, 755-758. https://doi.org/10.1086/303057

*Bahr, N., Christensen, C. A., & Bahr, M. (2005). Diversity of accuracy profiles for absolute pitch recognition. *Psychology of Music*, *33*, 58-93. https://doi.org/10.1177/0305735605048014

*Bairnsfather, J. E., Osborne, M. S., Martin, C., Mosing, M. A., & Wilson, S. J. (2022). Use of explicit priming to phenotype absolute pitch ability. *PLoS ONE*, *17*, Article e0273828. https://doi.org/10.1371/journal.pone.0273828

*Bairnsfather, J. E., Ullén, F., Osborne, M. S., Wilson, S. J., & Mosing, M. A. (2022). Investigating the relationship between childhood music practice and pitch-naming ability in professional musicians and a population-based twin sample. *Twin Research and Human Genetics*, *25*, 140-148. https://doi.org/10.1017/thg.2022.29

*Barnea, A., Granot, R., & Pratt, H. (1994). Absolute pitch - Electrophysiological evidence. *International Journal of Psychophysiology*, *16*, 29-38. https://doi.org/10.1016/0167-8760(94)90039-6

*Behroozmand, R., Ibrahim, N., Korzyukov, O., Robin, D. A., & Larson, C. R. (2014). Left-hemisphere activation is associated with enhanced vocal pitch error detection in musicians with absolute pitch. *Brain and Cognition*, *84*, 97-108. https://doi.org/10.1016/j.bandc.2013.11.007

*Benassi-Werke, M. E., Queiroz, M., Araújo, R. S., Bueno, O. F. A., & Oliveira, M. G. M. (2012). Musicians' working memory for tones, words, and pseudowords. *Quarterly Journal of Experimental Psychology*, *65*, 1161-1171. https://doi.org/10.1080/17470218.2011.644799

*Benner, J., Reinhardt, J., Christiner, M., Wengenroth, M., Stippich, C., Schneider, P., & Blatow, M. (2023). Temporal hierarchy of cortical responses reflects core-belt-parabelt organization of auditory cortex in musicians. *Cerebral Cortex*, *33*, 7044-7060. https://doi.org/10.1093/cercor/bhad020

*Bermudez, P., Lerch, J. P., Evans, A. C., & Zatorre, R. J. (2009). Neuroanatomical correlates of musicianship as revealed by cortical thickness and voxel-based morphometry. *Cerebral Cortex*, *19*, 1583-1596. https://doi.org/10.1093/cercor/bhn196

*Bermudez, P., & Zatorre, R. J. (2009). A distribution of absolute pitch ability as revealed by computerized testing. *Music Perception*, *27*, 89-101. https://doi.org/10.1525/mp.2009.27.2.89

Bittrich, K., Schlemmer, K., & Blankenberger, S. (2015). The impact of simple pair-association in the acquisition of absolute pitch: A training study with adult nonmusicians. *Music Perception*, *32*, 493-506. https://doi.org/10.1525/mp.2015.32.5.493

Boggs, L. P. (1907). Studies in absolute pitch. *The American Journal of Psychology*, *18*, 194-205. https://doi.org/10.2307/1412413

Borenstein, M., Hedges, L. V., Higgins, J. P. T., & Rothstein, H. R. (2021). *Introduction to meta-analysis* (2nd ed.). John Wiley & Sons. https://doi.org/10.1002/9780470743386

Brady, P. T. (1970). Fixed-scale mechanism of absolute pitch. *Journal of the Acoustical Society of America*, *48*, 883-887. https://doi.org/10.1121/1.1912227

*Brancucci, A., di Nuzzo, M., & Tommasi, L. (2009). Opposite hemispheric asymmetries for pitch identification in absolute pitch and non-absolute pitch musicians. *Neuropsychologia*, *47*, 2937-2941. https://doi.org/10.1016/j.neuropsychologia.2009.06.021

*Brancucci, A., Dipinto, R., Mosesso, I., & Tommasi, L. (2009). Vowel identity between note labels confuses pitch identification in non-absolute pitch possessors. *PLoS ONE*, *4*, Article e6327. https://doi.org/10.1371/journal.pone.0006327

*Brauchli, C., Leipold, S., & Jancke, L. (2020). Diminished large-scale functional brain networks in absolute pitch during the perception of naturalistic music and audiobooks. *NeuroImage*, *216*, Article 116513. https://doi.org/10.1016/j.neuroimage.2019.116513

*Brauchli, C., Leipold, S., & Jäncke, L. (2019). Univariate and multivariate analyses of functional networks in absolute pitch. *NeuroImage*, *189*, 241-247. https://doi.org/10.1016/j.neuroimage.2019.01.021

*Brown, W. A., Cammuso, K., Sachs, H., Winklosky, B., Mullane, J., Bernier, R., Svenson, S., Arin, D., Rosen-Sheidley, B., & Folstein, S. E. (2003). Autism-related language, personality, and cognition in people with absolute pitch: Results of a preliminary study. *Journal of Autism and Developmental Disorders*, *33*, 163-167. https://doi.org/10.1023/A:1022987309913

*Brown, W. A., Sachs, H., Cammuso, K., & Folstein, S. E. (2002). Early music training and absolute pitch. *Music Perception*, *19*, 595-597. https://doi.org/10.1525/mp.2002.19.4.595

*Burkhard, A., Elmer, S., & Jäncke, L. (2019). Early tone categorization in absolute pitch musicians is subserved by the right-sided perisylvian brain. *Scientific Reports*, *9*, Article 1419. https://doi.org/10.1038/s41598-018-38273-0

*Burkhard, A., Hänggi, J., Elmer, S., & Jäncke, L. (2020). The importance of the fibre tracts connecting the planum temporale in absolute pitch possessors. *NeuroImage*, *211*, Article 116590. https://doi.org/10.1016/j.neuroimage.2020.116590

*Burnham, D., Brooker, R., & Reid, A. (2015). The effects of absolute pitch ability and musical training on lexical tone perception. *Psychology of Music*, *43*, 881-897. https://doi.org/10.1177/0305735614546359

*Burns, E. M., & Campbell, S. L. (1994). Frequency and frequency-ratio resolution by possessors of absolute and relative pitch: Examples of categorical perception? *Journal of the Acoustical Society of America*, *96*, 2704-2719. https://doi.org/10.1121/1.411447

*Chavarria-Soley, G. (2016). Absolute pitch in Costa Rica: Distribution of pitch identification ability and implications for its genetic basis. *Journal of the Acoustical Society of America*, *140*, 891-897. https://doi.org/10.1121/1.4960569

*Coll, S. Y., Vuichoud, N., Grandjean, D., & James, C. E. (2019). Electrical neuroimaging of music processing in pianists with and without true absolute pitch. *Frontiers in Neuroscience*, *13*, Article 142. https://doi.org/10.3389/fnins.2019.00142

*Crummer, G. C., Walton, J. P., Wayman, J. W., Hantz, E. C., & Frisina, R. D. (1994). Neural processing of musical timbre by musicians, nonmusicians, and musicians possessing absolute pitch. *Journal of the Acoustical Society of America*, *95*, 2720-2727. https://doi.org/10.1121/1.409840

Cuddy, L. L. (1968). Practice effects in the absolute judgment of pitch. *Journal of the Acoustical Society of America*, *43*, 1069-1076. https://doi.org/10.1121/1.1910941

Cuddy, L. L. (1970). Training the absolute identification of pitch. *Perception & Psychophysics*, *8*, 265-269. https://doi.org/10.3758/BF03212589

*Deutsch, D., & Dooley, K. (2013). Absolute pitch is associated with a large auditory digit span: A clue to its genesis. *Journal of the Acoustical Society of America*, *133*, 1859-1861. https://doi.org/10.1121/1.4792217

*Deutsch, D., Dooley, K., Henthorn, T., & Head, B. (2009). Absolute pitch among students in an American music conservatory: Association with tone language fluency. *Journal of the Acoustical Society of America*, *125*, 2398-2403. https://doi.org/10.1121/1.3081389

*Deutsch, D., Edelstein, M., Dooley, K., & Henthorn, T. (2021). Absolute pitch is disrupted by a memory illusion. *Journal of the Acoustical Society of America*, *149*, 2829-2835. https://doi.org/10.1121/10.0004776

*Deutsch, D., Henthorn, T., Marvin, E., & HongShuai, X. (2006). Absolute pitch among American and Chinese conservatory students: Prevalence differences, and evidence for a speech-related critical period. *Journal of the Acoustical Society of America*, *119*, 719-722. https://doi.org/10.1121/1.2151799

*Deutsch, D., Li, X., & Shen, J. (2013). Absolute pitch among students at the Shanghai Conservatory of Music: A large-scale direct-test study. *Journal of the Acoustical Society of America*, *134*, 3853-3859. https://doi.org/10.1121/1.4824450

*Di Giuseppe Germano, N., Cogo-Moreira, H., Coutinho-Lourenço, F., & Bortz, G. (2021). A new approach to measuring absolute pitch on a psychometric theory of isolated pitch perception: Is it disentangling specific groups or capturing a continuous ability? *PLoS ONE*, *16*, Article e0247473. https://doi.org/10.1371/journal.pone.0247473

*Dohn, A., Garza-Villarreal, E. A., Chakravarty, M. M., Hansen, M., Lerch, J. P., & Vuust, P. (2015). Gray-and white-matter anatomy of absolute pitch possessors. *Cerebral Cortex*, *25*, 1379-1388. https://doi.org/10.1093/cercor/bht334

*Dohn, A., Garza-Villarreal, E. A., Heaton, P., & Vuust, P. (2012). Do musicians with perfect pitch have more autism traits than musicians without perfect pitch? an empirical study. *PLoS ONE*, *7*, Article e37961. https://doi.org/10.1371/journal.pone.0037961

*Dohn, A., Garza-Villarreal, E. A., Ribe, L. R., Wallentin, M., & Vuust, P. (2014). Musical activity tunes up absolute pitch ability. *Music Perception*, *31*, 359-371. https://doi.org/10.1525/MP.2014.31.4.359

*Dooley, K., & Deutsch, D. (2010). Absolute pitch correlates with high performance on musical dictation. *Journal of the Acoustical Society of America*, *128*, 890-893. https://doi.org/10.1121/1.3458848

*Dooley, K., & Deutsch, D. (2011). Absolute pitch correlates with high performance on interval naming tasks. *Journal of the Acoustical Society of America*, *130*, 4097-4104. https://doi.org/10.1121/1.3652861

*Elmer, S., Rogenmoser, L., Kuhnis, J., & Jäncke, L. (2015). Bridging the gap between perceptual and cognitive perspectives on absolute pitch. *Journal of Neuroscience*, *35*, 366-371. https://doi.org/10.1523/JNEUROSCI.3009-14.2015

*Elmer, S., Sollberger, S., Meyer, M., & Jäncke, L. (2013). An empirical reevaluation of absolute pitch: Behavioral and electrophysiological measurements. *Journal of Cognitive Neuroscience*, *25*, 1736-1753. https://doi.org/10.1162/jocn_a_00410

*Fujisaki, W., & Kashino, M. (2002). The basic hearing abilities of absolute pitch possessors. *Acoustical Science and Technology*, *23*, 77-83. https://doi.org/10.1250/ast.23.77

*Fujisaki, W., & Kashino, M. (2005). Contributions of temporal and place cues in pitch perception in absolute pitch possessors. *Perception and Psychophysics*, *67*, 315-323. https://doi.org/10.3758/BF03206494

Gordon, M., & Lumley, T. (2024). *forestplot: Advanced forest plot Using 'grid' graphics*. R package version 3.1.3, https://github.com/gforge/forestplot

*Greber, M., & Jäncke, L. (2020). Suppression of pitch labeling: No evidence for an impact of absolute pitch on behavioral and neurophysiological measures of cognitive inhibition in an auditory go/nogo task. *Frontiers in Human Neuroscience*, *14*, Article 585505. https://doi.org/10.3389/fnhum.2020.585505

*Greber, M., Klein, C., Leipold, S., Sele, S., & Jäncke, L. (2020). Heterogeneity of EEG resting-state brain networks in absolute pitch. *International Journal of Psychophysiology*, *157*, 11-22. https://doi.org/10.1016/j.ijpsycho.2020.07.007

*Greber, M., Rogenmoser, L., Elmer, S., & Jäncke, L. (2018). Electrophysiological correlates of absolute pitch in a passive auditory oddball paradigm: A direct replication attempt. *eNeuro*, *5*, Article e0333. https://doi.org/10.1523/ENEURO.0333-18.2018

*Gregersen, P. K., Kowalsky, E., Kohn, N., & Marvin, E. W. (1999). Absolute pitch: Prevalence, ethnic variation, and estimation of the genetic component. *American Journal of Human Genetics*, *65*(3), 911-913. https://doi.org/10.1086/302541

*Gregersen, P. K., Kowalsky, E., Kohn, N., & Marvin, E. W. (2001). Letter to the editor: Early childhood music education and predisposition to absolute pitch: Teasing apart genes and environment. *American Journal of Medical Genetics*, *98*, 280-282. https://doi.org/10.1002/1096-8628(20010122)98:3<280::AID-AJMG1083>3.0.CO;2-6

Gregersen, P. K., Kowalsky, E., Lee, A., Baron-Cohen, S., Fisher, S. E., Asher, J. E., Ballard, D., Freudenberg, J., & Li, W. (2013). Absolute pitch exhibits phenotypic and genetic overlap with synesthesia. *Human Molecular Genetics*, *22*, 2097-2104. https://doi.org/10.1093/hmg/ddt059

*Gruhn, W., Ristmägi, R., Schneider, P., D'Souza, A., & Kiilu, K. (2018). How stable is pitch labeling accuracy in absolute pitch possessors? *Empirical Musicology Review*, *13*, 110-123. https://doi.org/10.18061/emr.v13i3-4.6637

*Hantz, E. C., Crummer, G. C., Wayman, J. W., Walton, J. P., & Frisina, R. D. (1992). Effects of musical training and absolute pitch on the neural processing of melodic intervals: A P3 event-related potential study. *Music Perception*, *10*, 25-42. https://doi.org/10.2307/40285536

*Hantz, E. C., Kreilick, K. G., Braveman, A. L., & Swartz, K. P. (1995). Effects of musical training and absolute pitch on a pitch memory task: An event-related potential study. *Psychomusicology*, *14*, 53-76. https://doi.org/10.1037/h0094091

*Hantz, E. C., Kreilick, K. G., Marvin, E. W., & Chapman, R. M. (1997). Absolute pitch and sex affect event-related potential activity for a melodic interval discrimination task. *Journal of the Acoustical Society of America*, *102*, 451-460. https://doi.org/10.1121/1.419718

Heald, S. L. M., Van Hedger, S. C., & Nusbaum, H. C. (2014). Auditory category knowledge in experts and novices. *Frontiers in Neuroscience*, Article 260. https://doi.org/10.3389/fnins.2014.00260

Heaton, P., Davis, R. E., & Happe, F. G. (2008). Research note: Exceptional absolute pitch perception for spoken words in an able adult with autism. *Neuropsychologia*, *46*, 2095-2098. https://doi.org/10.1016/j.neuropsychologia.2008.02.006

*Hedger, S. C., Heald, S. L. M., & Nusbaum, H. C. (2013). Absolute pitch may not be so absolute. *Psychological Science*, *24*, 1496-1502. https://doi.org/10.1177/0956797612473310

*Hirata, Y., Kuriki, S., & Pantev, C. (1999). Musicians with absolute pitch show distinct neural activities in the auditory cortex. *NeuroReport*, *10*, 999-1002. https://doi.org/10.1097/00001756-199904060-00019

*Hirose, H., Kubota, M., Kimura, I., Ohsawa, M., Yumoto, M., & Sakakihara, Y. (2002). People with absolute pitch process tones with producing P300. *Neuroscience Letters*, *330*, 247-250. https://doi.org/10.1016/S0304-3940(02)00812-1

*Hirose, H., Kubota, M., Kimura, I., Yumoto, M., & Sakakihara, Y. (2004). N100m in adults possessing absolute pitch. *NeuroReport*, *15*, 1383-1386. https://doi.org/10.1097/01.wnr.0000132921.90118.c4

*Hirose, H., Kubota, M., Kimura, I., Yumoto, M., & Sakakihara, Y. (2005). Increased right auditory cortex activity in absolute pitch possessors. *NeuroReport*, *16*, 1775-1779. https://doi.org/10.1097/01.wnr.0000183906.00526.51

*Hou, J., Chen, C., Dong, Q., Prabhakaran, V., & Nair, V. A. (2021). Superior pitch identification ability is associated with better mental rotation performance. *Musicae Scientiae*, 1-20. https://doi.org/10.1177/10298649211013409

*Hou, J., Chen, C., O’Boyle, M. W., & Dong, Q. (2023). Superior pitch identification ability revealed by cortical complexity measures in nonmusicians. *Psychology of Music*, *51*, 820-837. https://doi.org/10.1177/03057356221110634

*Hou, J., Chen, C., Wang, Y., Liu, Y., He, Q., Li, J., & Dong, Q. (2014). Superior pitch identification ability is associated with better executive functions. *Psychomusicology*, *24*, 136-146. https://doi.org/10.1037/a0036963

*Hsieh, I. H., & Saberi, K. (2007). Temporal integration in absolute identification of musical pitch. *Hearing Research*, *233*, 108-116. https://doi.org/10.1016/j.heares.2007.08.005

*Hsieh, I. H., & Saberi, K. (2008a). Dissociation of procedural and semantic memory in absolute-pitch processing. *Hearing Research*, *240*, 73-79. https://doi.org/10.1016/j.heares.2008.01.017

*Hsieh, I. H., & Saberi, K. (2008b). Language-selective interference with long-term memory for musical pitch. *Acta Acustica united with Acustica*, *94*, 588-593. https://doi.org/10.3813/AAA.918068

*Hsieh, I. H., & Saberi, K. (2009). Virtual pitch extraction from harmonic structures by absolute-pitch musicians. *Acoustical Physics*, *55*, 232-239. https://doi.org/10.1134/S1063771009020134

*Hsieh, I. H., Tseng, H. C., & Liu, J. W. (2022). Domain-specific hearing-in-noise performance is associated with absolute pitch proficiency. Scientific Reports, 12, Article 16344. https://doi.org/10.1038/s41598-022-20869-2

*Hutchins, S., Hutka, S., & Moreno, S. (2015). Symbolic and motor contributions to vocal imitation in absolute pitch. *Music Perception*, *32*, 254-265. https://doi.org/10.1525/MP.2015.32.3.254

*Hutka, S. A., & Alain, C. (2015). The effects of absolute pitch and tone language on pitch processing and encoding in musicians. *Music Perception*, *32*, 344-354. https://doi.org/10.1525/MP.2015.32.4.344

*Itoh, K., Miyazaki, K., & Nakada, T. (2003). Ear advantage and consonance of dichotic pitch intervals in absolute-pitch possessors. *Brain and Cognition*, *53*, 464-471. https://doi.org/10.1016/S0278-2626(03)00236-7

*Itoh, K., Suwazono, S., Arao, H., Miyazaki, K., & Nakada, T. (2005). Electrophysiological correlates of absolute pitch and relative pitch. *Cerebral Cortex*, *15*, 760-769. https://doi.org/10.1093/cercor/bhh177

*Jäncke, L., Langer, N., & Hänggi, J. (2012). Diminished whole-brain but enhanced peri-sylvian connectivity in absolute pitch musicians. *Journal of Cognitive Neuroscience*, *24*, 1447-1461. https://doi.org/10.1162/jocn_a_00227

Jiang, C.-M., Zhang, Q., Li, W.-J., & Yang, Y.-F. (2010). Influence of absolute pitch on music syntax processing. *Acta Psychologica Sinica*, *42*, 443-451. https://doi.org/10.3724/sp.j.1041.2010.00443

*Jiang, J., Hai, T., Man, D., & Zhou, L. (2020). Is absolute pitch associated with musical tension processing? *i-Perception*, *11*, 1-16. https://doi.org/10.1177/2041669520971655

*Kamiyama, K., Katahira, K., Abla, D., Hori, K., & Okanoya, K. (2010). Music playing and memory trace: Evidence from event-related potentials. *Neuroscience Research*, *67*, 334-340. https://doi.org/10.1016/j.neures.2010.04.007

*Keenan, J. P., Thangaraj, V., Halpern, A. R., & Schlaug, G. (2001). Absolute pitch and planum temporale. *NeuroImage*, *14*, 1402-1408. https://doi.org/10.1006/nimg.2001.0925

*Kim, S., Blake, R., Lee, M., & Kim, C. Y. (2017). Audio-visual interactions uniquely contribute to resolution of visual conflict in people possessing absolute pitch. *PLoS ONE*, *12*, Article e0175103. https://doi.org/10.1371/journal.pone.0175103

*Kim, S. G., & Knösche, T. R. (2016). Intracortical myelination in musicians with absolute pitch: Quantitative morphometry using 7-T MRI. *Human Brain Mapping*, *37*, 3486-3501. https://doi.org/10.1002/hbm.23254

*Kim, S. G., & Knösche, T. R. (2017). Resting state functional connectivity of the ventral auditory pathway in musicians with absolute pitch. *Human Brain Mapping*, *38*, 3899-3916. https://doi.org/10.1002/hbm.23637

Klein, M. E., Coles, M. G. H., & Donchin, E. (1984). People with absolute pitch process tones without producing a P300. *Science*, *223*, 1306-1309. https://doi.org/10.1126/science.223.4642.1306

*Lee, C. Y., & Hung, T. H. (2008). Identification of Mandarin tones by English-speaking musicians and nonmusicians. *Journal of the Acoustical Society of America*, *124*, 3235-3248. https://doi.org/10.1121/1.2990713

*Lee, C. Y., & Lee, Y. F. (2010). Perception of musical pitch and lexical tones by Mandarin-speaking musicians. *Journal of the Acoustical Society of America*, *127*, 481-490. https://doi.org/10.1121/1.3266683

*Lee, C. Y., Lee, Y. F., & Shr, C. L. (2011). Perception of musical and lexical tones by Taiwanese-speaking musicians. *Journal of the Acoustical Society of America*, *130*, 526-535. https://doi.org/10.1121/1.3596473

*Leipold, S., Brauchli, C., Greber, M., & Jäncke, L. (2019). Absolute and relative pitch processing in the human brain: Neural and behavioral evidence. *Brain Structure and Function*, *224*, 1723-1738. https://doi.org/10.1007/s00429-019-01872-2

*Leipold, S., Greber, M., Sele, S., & Jäncke, L. (2019). Neural patterns reveal single-trial information on absolute pitch and relative pitch perception. *NeuroImage*, *200*, 132-141. https://doi.org/10.1016/j.neuroimage.2019.06.030

*Leipold, S., Klein, C., & Jäncke, L. (2021). Musical expertise shapes functional and structural brain networks independent of absolute pitch ability. *The Journal of Neuroscience*, *41*, 2496-2511. https://doi.org/10.1523/JNEUROSCI.1985-20.2020

*Leipold, S., Oderbolz, C., Greber, M., & Jäncke, L. (2019). A reevaluation of the electrophysiological correlates of absolute pitch and relative pitch: No evidence for an absolute pitch-specific negativity. *International Journal of Psychophysiology*, *137*, 21-31. https://doi.org/10.1016/j.ijpsycho.2018.12.016 *Leite, R. B. C., Mota-Rolim, S. A., & Queiroz, C. M. T. (2016). Music proficiency and quantification of absolute pitch: A large-scale study among Brazilian musicians. *Frontiers in Neuroscience*, *10*, Article 447. https://doi.org/10.3389/fnins.2016.00447

*Leite Filho, C. A., Rocha-Muniz, C. N., Pereira, L. D., & Schochat, E. (2023). Auditory temporal resolution and backward masking in musicians with absolute pitch. *Frontiers in Neuroscience*, *17*, Article 1151776. https://doi.org/10.3389/fnins.2023.1151776

Levitin, D. J., & Zatorre, R. J. (2003). On the nature of early music training and absolute pitch: A reply to Brown, Sachs, Cammuso, and Folstein. *Music Perception*, *21*, 105-110. https://doi.org/10.1525/mp.2003.21.1.105

*Li, X. (2021). The effects of timbre on absolute pitch judgment. *Psychology of Music*, *49*, 704-717. https://doi.org/10.1177/0305735619893437

Linden, A. H., & Hönekopp, J. (2021). Heterogeneity of research results: A new perspective from which to assess and promote progress in psychological science. *Perspectives on Psychological Science, 16*, 358-376. https://doi.org/10.1177/1745691620964193

Lockhead, G. R., & Byrd, R. (1981). Practically perfect pitch. *Journal of the Acoustical Society of America*, *70*, 387-389. https://doi.org/10.1121/1.386773

*Loui, P., Li, H. C., Hohmann, A., & Schlaug, G. (2011). Enhanced cortical connectivity in absolute pitch musicians: A model for local hyperconnectivity. *Journal of Cognitive Neuroscience*, *23*, 1015-1026. https://doi.org/10.1162/jocn.2010.21500

*Loui, P., Zamm, A., & Schlaug, G. (2012). Enhanced functional networks in absolute pitch. *NeuroImage*, *63*, 632-640. https://doi.org/10.1016/j.neuroimage.2012.07.030

*Luders, E., Gaser, C., Jancke, L., & Schlaug, G. (2004). A voxel-based approach to gray matter asymmetries. *NeuroImage*, *22*, 656-664. https://doi.org/10.1016/j.neuroimage.2004.01.032

*Maeshima, H., Hosoda, C., Okanoya, K., & Nakai, T. (2018). Reduced gamma-aminobutyric acid in the superior temporal gyrus is associated with absolute pitch. *NeuroReport*, *29*, 1487-1491. https://doi.org/10.1097/wnr.0000000000001137

*Maggu, A. R., Lau, J. C. Y., Waye, M. M. Y., & Wong, P. C. M. (2021). Combination of absolute pitch and tone language experience enhances lexical tone perception. *Scientific Reports*, *11*, Article 1485. https://doi.org/10.1038/s41598-020-80260-x

*Masataka, N. (2011). Enhancement of speech-relevant auditory acuity in absolute pitch possessors. *Frontiers in Psychology*, *2*, Article 101. https://doi.org/10.3389/fpsyg.2011.00101

*Matsuda, A., Hara, K., Watanabe, S., Matsuura, M., Ohta, K., & Matsushima, E. (2013). Pre-attentive auditory processing of non-scale pitch in absolute pitch possessors. *Neuroscience Letters*, *548*, 155-158. https://doi.org/10.1016/j.neulet.2013.05.049

*Matsuda, M., Igarashi, H., & Itoh, K. (2019). Auditory T-complex reveals reduced neural activities in the right auditory cortex in musicians with absolute pitch. *Frontiers in Neuroscience*, *13*, Article 809. https://doi.org/10.3389/fnins.2019.00809

Matsunaga, R., & Abe, J.-I. (2005). Cues for key perception of a melody: Pitch set alone? *Music Perception*, *23*, 153-164. https://doi.org/10.1525/mp.2005.23.2.153

*McKetton, L., DeSimone, K., & Schneider, K. A. (2019). Larger auditory cortical area and broader frequency tuning underlie absolute pitch. *Journal of Neuroscience*, *39*, 2930-2937. https://doi.org/10.1523/JNEUROSCI.1532-18.2019

*McKetton, L., Purcell, D., Stone, V., Grahn, J., & Bergevin, C. (2018). No otoacoustic evidence for a peripheral basis of absolute pitch. *Hearing Research*, *370*, 201-208. https://doi.org/10.1016/j.heares.2018.08.001

*McLachlan, N. M., Marco, D. J. T., & Wilson, S. J. (2013). Pitch and plasticity: Insights from the pitch matching of chords by musicians with absolute and relative pitch. *Brain Sciences*, *3*, 1615-1634. https://doi.org/10.3390/brainsci3041615

*Miller, L. K., & Clausen, H. (1997). Pitch identification in children and adults: Naming and discrimination. *Psychology of Music*, *25*, 4-17. https://doi.org/10.1177/0305735697251002

*Miskiewicz, A., & Rakowski, A. (2012). A psychophysical pitch function determined by absolute magnitude estimation and its relation to the musical pitch scale. *Journal of the Acoustical Society of America*, *131*, 987-992. https://doi.org/10.1121/1.3651094

*Mito, H. (2003). Performance at a transposed keyboard by possessor and non-possessor of absolute pitch. *Bulletin of the Council for Research in Music Education*, *157*, 18-23.

Miyazaki, K. (1988). Musical pitch identification by absolute pitch possessors. *Perception & Psychophysics*, *44*, 504-512. https://doi.org/10.3758/bf03207484

Miyazaki, K. (1989). Absolute pitch identification: Effects of timbre and pitch region. *Music Perception*, *7*, 1-14. https://doi.org/10.2307/40285445

Miyazaki, K. (1990). The speed of musical pitch identification by absolute-pitch possessors. *Music Perception*, *8*, 177-188. https://doi.org/10.2307/40285495

*Miyazaki, K. (1992). Perception of musical intervals by absolute pitch possessors. *Music Perception*, *9*, 413-426. https://doi.org/10.2307/40285562

*Miyazaki, K. (1993). Absolute pitch as an inability: Identification of musical intervals in a tonal context. *Music Perception*, *11*, 55-71. https://doi.org/10.2307/40285599

*Miyazaki, K. (1995). Perception of relative pitch with different references: Some absolute-pitch listeners can't tell musical interval names. *Perception & Psychophysics*, *57*, 962-970. https://doi.org/10.3758/BF03205455

Miyazaki, K. (2004a). How well do we understand absolute pitch? *Acoustical Science and Technology*, *25*, 426-432. https://doi.org/10.1250/ast.25.426

*Miyazaki, K. (2004b). Recognition of transposed melodies by absolute-pitch possessors. *Japanese Psychological Research*, *46*, 270-282. https://doi.org/10.1111/j.1468-5584.2004.00260.x

*Miyazaki, K., Makomaska, S., & Rakowski, A. (2012). Prevalence of absolute pitch: A comparison between Japanese and Polish music students. *Journal of the Acoustical Society of America*, *132*, 3484-3493. https://doi.org/10.1121/1.4756956

*Miyazaki, K., & Rakowski, A. (2002). Recognition of notated melodies by possessors and nonpossessors of absolute pitch. *Perception & Psychophysics*, *64*, 1337-1345. https://doi.org/10.3758/BF03194776

*Miyazaki, K., Rakowski, A., Makomaska, S., Jiang, C., Tsuzaki, M., Oxenham, A. J., Ellis, G., & Lipscomb, S. D. (2018). Absolute pitch and relative pitch in music students in the east and the west: Implications for aural-skills education. *Music Perception*, *36*, 135-155. https://doi.org/10.1525/mp.2018.36.2.135

*Ngan, V. S. H., Cheung, L. Y. T., Ng, H. T. Y., Yip, K. H. M., Wong, Y. K., & Wong, A. C. N. (2023). An early perceptual locus of absolute pitch. *Psychophysiology*, *60*, Article e14170. https://doi.org/10.1111/psyp.14170

*Oechslin, M. S., Imfeld, A., Loenneker, T., Meyer, M., & Jäncke, L. (2010). The plasticity of the superior longitudinal fasciculus as a function of musical expertise: A diffusion tensor imaging study. *Frontiers in Human Neuroscience*, *3*, Article 76. https://doi.org/10.3389/neuro.09.076.2009

*Oechslin, M. S., Meyer, M., & Jäncke, L. (2010). Absolute pitch-functional evidence of speech-relevant auditory acuity. *Cerebral Cortex*, *20*, 447-455. https://doi.org/10.1093/cercor/bhp113

*Ohnishi, T., Matsuda, H., Asada, T., Aruga, M., Hirakata, M., Nishikawa, M., Katoh, A., & Imabayashi, E. (2001). Functional anatomy of musical perception in musicians. *Cerebral Cortex*, *11*, 754-760. https://doi.org/10.1093/cercor/11.8.754

*Pantev, C., Oostenveld, R., Engelien, A., Ross, B., Roberts, L. E., & Hoke, M. (1998). Increased auditory cortical representation in musicians. *Nature*, *392*, 811-814. https://doi.org/10.1038/33918

*Parkinson, A. L., Behroozmand, R., Ibrahim, N., Korzyukov, O., Larson, C. R., & Robin, D. A. (2014). Effective connectivity associated with auditory error detection in musicians with absolute pitch. *Frontiers in Neuroscience*, Article 46. https://doi.org/10.3389/fnins.2014.00046

*Peng, G., Deutsch, D., Henthorn, T., Su, D., & Wang, W. S. Y. (2013). Language experience influences non-linguistic pitch perception. *Journal of Chinese Linguistics*, *41*, 447-467.

Pfordresher, P., & Kobrina, A. (2017). Sensitivity to meter in auditory feedback during music performance. *Psychomusicology*, *27*, 54-62. https://doi.org/10.1037/PMU0000166

Polderman, T. J. C., Benyamin, B., de Leeuw, C. A., Sullivan, P. F., van Bochoven, A., Visscher, P. M., & Posthuma, D. (2015). Meta-analysis of the heritability of human traits based on fifty years of twin studies. *Nature Genetics, 47*, 702-709. https://doi.org/10.1038/ng.3285

Profita, J., & Bidder, T. G. (1988). Perfect pitch. *American Journal of Medical Genetics*, *29*, 763-771. https://doi.org/10.1002/ajmg.1320290405

*Rakowski, A., & Rogowski, P. (2007). Experiments on long-term and short-term memory for pitch in musicians. *Archives of Acoustics*, *32*, 815-826.

*Rakowski, A., & Rogowski, P. (2011). Absolute pitch and its frequency range. *Archives of Acoustics*, *36*, 251-266. https://doi.org/10.2478/v10168-011-0020-1

*Reis, K. S., Heald, S. L. M., Veillette, J. P., Van Hedger, S. C., & Nusbaum, H. C. (2021). Individual differences in human frequency-following response predict pitch labeling ability. *Scientific Reports*, *11*, Article 14290. https://doi.org/10.1038/s41598-021-93312-7

*Remijn, G. B., Teramachi, M., & Ueda, K. (2024). Auditory ensemble perception (summary statistics) for music scale tones by listeners with and without absolute pitch. *Auditory Perception & Cognition, 7,* 163–178. https://doi.org/10.1080/25742442.2024.2310460

*Renninger, L. B., Granot, R. I., & Donchin, E. (2003). Absolute pitch and the P300 component of the event-related potential: An exploration of variables that may account for individual differences. *Music Perception*, *20*, 357-382. https://doi.org/10.1525/mp.2003.20.4.357

Revelle, W. (2024). *psych: Procedures for psychological, psychometric, and personality Research*. Northwestern University, Evanston, Illinois. R package version 2.3.6, https://CRAN.R-project.org/package=psych.

*Rogenmoser, L., Arnicane, A., Jäncke, L., & Elmer, S. (2021). The left dorsal stream causally mediates the tone labeling in absolute pitch. *Annals of the New York Academy of Sciences*, *1500*, 122-133. https://doi.org/10.1111/nyas.14616

*Rogenmoser, L., Elmer, S., & Jäncke, L. (2015). Absolute pitch: Evidence for early cognitive facilitation during passive listening as revealed by reduced P3a amplitudes. *Journal of Cognitive Neuroscience*, *27*, 623-637. https://doi.org/10.1162/jocn_a_00708

*Rogenmoser, L., Li, H. C., Jäncke, L., & Schlaug, G. (2021). Auditory aversion in absolute pitch possessors. *Cortex*, *135*, 285-297. https://dx.doi.org/10.1016/j.cortex.2020.11.020

*Rogowski, P., & Rakowski, A. (2010). Pitch strength of residual sounds estimated through chroma recognition by absolute-pitch possessors. *Archives of Acoustics*, *35*, 331-347. https://doi.org/10.2478/v10168-010-0028-y

*Ross, D. A., Olson, I. R., & Gore, J. C. (2003). Absolute pitch does not depend on early musical training. In *Annals of the New York Academy of Sciences* (Vol. 999, pp. 522-526).

*Ross, D. A., Olson, I. R., Marks, L. E., & Gore, J. C. (2004). A nonmusical paradigm for identifying absolute pitch possessors. *Journal of the Acoustical Society of America*, *116*, 1793-1799. https://doi.org/10.1121/1.1758973

Ruscio, J., Haslam, N., & Ruscio, A. M. (2006*). Introduction to the taxometric method: A practical guide.* Lawrence Erlbaum Associates Publishers.

Russo, F. A., Windell, D. L., & Cuddy, L. L. (2003). Learning the “special note”: Evidence for a critical period for absolute pitch acquisition. *Music Perception*, *21*, 119-127. https://doi.org/10.1525/mp.2003.21.1.119

Sakakibara, A. (2014). A longitudinal study of the process of acquiring absolute pitch: A practical report of training with the 'chord identification method'. *Psychology of Music*, *42*, 86-111. https://doi.org/10.1177/0305735612463948

Sarkar, D. (2008). *Lattice: Multivariate data visualization with R*. Springer, New York. ISBN 978-0-387-75968-5, <http://lmdvr.r-forge.r-project.org>

*Schlaug, G., Jäncke, L., Huang, Y., & Steinmetz, H. (1995). In vivo evidence of structural brain asymmetry in musicians. *Science*, *267*, 699-701. https://doi.org/10.1126/science.7839149

*Schlemmer, K. B., Kulke, F., Kuchinke, L., & Van Der Meer, E. (2005). Absolute pitch and pupillary response: Effects of timbre and key color. *Psychophysiology*, *42*, 465-472. https://doi.org/10.1111/j.1469-8986.2005.00306.x

*Schulze, K., Gaab, N., & Schlaug, G. (2009). Perceiving pitch absolutely: Comparing absolute and relative pitch possessors in a pitch memory task. *BMC Neuroscience*, *10*, Article 1471. https://doi.org/10.1186/1471-2202-10-106

*Schulze, K., Mueller, K., & Koelsch, S. (2013). Auditory stroop and absolute pitch: An fMRI study. *Human Brain Mapping*, *34*, 1579-1590. https://doi.org/10.1002/hbm.22010

Seashore, C. E. (1939). The psychology of music. XX. Musical inheritance. *Music Educators Journal*, *25*(6), 21. https://doi.org/10.2307/3385396

Seashore, C. E. (1940). The psychology of music. XXVI. Acquired pitch vs. absolute pitch. *Music Educators Journal*, *26*(6), 18. https://doi.org/10.2307/3385750

**Sharma, V. V., Thaut, M., Russo, F., & Alain, C. (2019). Absolute pitch and musical expertise modulate neuro-electric and behavioral responses in an auditory Stroop paradigm. *Frontiers in Neuroscience*, *13*, Article 932. https://doi.org/10.3389/fnins.2019.00932

*Sharma, V. V., Thaut, M., Russo, F. A., & Alain, C. (2023). Absolute pitch: Neurophysiological evidence for early brain activity in prefrontal cortex. *Cerebral Cortex*, *33*, 6465-6473. https://doi.org/10.1093/cercor/bhac517

*Shigeno, S. (1993). The interdependence of pitch and temporal judgments by absolute pitch possessors. *Perception & Psychophysics*, *54*, 682-692. https://doi.org/10.3758/bf03211792

Takeuchi, A. H., & Hulse, S. H. (1993). Absolute pitch. *Psychological Bulletin*, *113*, 345-361. https://doi.org/10.1037/0033-2909.113.2.345

*Temperley, D., & Marvin, E. W. (2008). Pitch-class distribution and the identification of key. *Music Perception*, *25*, 193-212. https://doi.org/10.1525/mp.2008.25.3.193

*Tervaniemi, M., Paavilainen, P., Sams, M., Näätänen, R., & Alho, K. (1993). Absolute pitch and event-related brain potentials. *Music Perception*, *10*, 305-316. https://doi.org/10.2307/40285572

*Theusch, E., Basu, A., & Gitschier, J. (2009). Genome-wide study of families with absolute pitch reveals linkage to 8q24.21 and locus heterogeneity. *American Journal of Human Genetics*, *85*, 112-119. https://doi.org/10.1016/j.ajhg.2009.06.010

*Theusch, E., & Gitschier, J. (2011). Absolute pitch twin study and segregation analysis. *Twin Research and Human Genetics*, *14*, 173-178. https://doi.org/10.1375/twin.14.2.173

*Tseng, H. C., & Hsieh, I. H. (2024). Effects of absolute pitch on brain activation and functional connectivity during hearing-in-noise perception. *Cortex*, *174*, 1-18. https://doi.org/10.1016/j.cortex.2024.02.011

*Van Hedger, S. C., Heald, S. L., Uddin, S., & Nusbaum, H. C. (2018). A note by any other name: Intonation context rapidly changes absolute note judgments. *Journal of Experimental Psychology: Human Perception and Performance*, *44*, 1268-1282. https://doi.org/10.1037/xhp0000536

*Van Hedger, S. C., Heald, S. L. M., & Nusbaum, H. C. (2016). What the [bleep]? Enhanced absolute pitch memory for a 1000 Hz sine tone. *Cognition*, *154*, 139-150. https://doi.org/10.1016/j.cognition.2016.06.001

*Van Hedger, S. C., & Nusbaum, H. C. (2018). Individual differences in absolute pitch performance: Contributions of working memory, musical expertise, and tonal language background. *Acta Psychologica*, *191*, 251-260. https://doi.org/10.1016/j.actpsy.2018.10.007

Van Hedger, S. C., Heald, S. L. M., & Nusbaum, H. C. (2019). Absolute pitch can be learned by some adults. *PLoS ONE*, *14*, Article e0223047. https://doi.org/10.1371/journal.pone.0223047

*Van Hedger, S. C., Veillette, J., Heald, S. L. M., & Nusbaum, H. C. (2020). Revisiting discrete versus continuous models of human behavior: The case of absolute pitch. *PLoS ONE*, *15*, Article e0244308. https://dx.doi.org/10.1371/journal.pone.0244308

*Vanzella, P., Braun Janzen, T., Oliveira, G. A. D., Sato, J. R., & Fraga, F. J. (2023). Perception and identification of tones in different timbres: Using pupil diameter to investigate absolute pitch ability. *Psychology of Music*, *51*, 226-243. https://doi.org/10.1177/03057356221091598

*Vanzella, P., & Schellenberg, E. G. (2010). Absolute pitch: Effects of timbre on note-naming ability. *PLoS ONE*, *5*, Article e15449. https://doi.org/10.1371/journal.pone.0015449

Vitouch, O. (2003). Absolutist models of absolute pitch are absolutely misleading. *Music Perception*, *21*, 111-117. https://doi.org/10.1525/mp.2003.21.1.111

Ward, W. D. (1999). Absolute pitch. In D. Deutsch (Ed.), *The Psychology of Music* (2nd ed., pp. 265-298). Elsevier.

Ward, W. D., & Burns, E. M. (1982). Absolute pitch. In D. Deutsch (Ed.), *The Psychology of Music* (pp. 431-451). Academic Press.

*Wayman, J. W., Frisina, R. D., Walton, J. P., Hantz, E. C., & Crummer, G. C. (1992). Effects of musical training and absolute pitch ability on event-related activity in response to sine tones. *Journal of the Acoustical Society of America*, *91*, 3527-3531. https://doi.org/10.1121/1.402841

*Weisman, R. G., Balkwill, L.-L., Hoeschele, M., Moscicki, M. K., & Sturdy, C. B. (2012). Identifying absolute pitch possessors without using a note-naming task. *Psychomusicology*, *22*, 46-54. https://doi.org/10.1037/a0028940

*Weiss, M. W., Vanzella, P., Schellenberg, E. G., & Trehub, S. E. (2015). Pianists exhibit enhanced memory for vocal melodies but not piano melodies. *The Quarterly Journal of Experimental Psychology*, *68*, 866-877. https://doi.org/10.1080/17470218.2015.1020818

*Wengenroth, M., Blatow, M., Heinecke, A., Reinhardt, J., Stippich, C., Hofmann, E., & Schneider, P. (2014). Increased volume and function of right auditory cortex as a marker for absolute pitch. *Cerebral Cortex*, *24*, 1127-1137. https://doi.org/10.1093/cercor/bhs391

*Wenhart, T., & Altenmüller, E. (2019). A tendency towards details? Inconsistent results on auditory and visual local-to-global processing in absolute pitch musicians. *Frontiers in Psychology*, *10*, Article 31. https://doi.org/10.3389/fpsyg.2019.00031

*Wenhart, T., Bethlehem, R. A. I., Baron-Cohen, S., & Altenmüller, E. (2019). Autistic traits, resting-state connectivity, and absolute pitch in professional musicians: Shared and distinct neural features. *Molecular Autism*, *10*, Article 20. https://doi.org/10.1186/s13229-019-0272-6

*Wenhart, T., Hwang, Y. Y., & Altenmüller, E. (2019). Enhanced auditory disembedding in an interleaved melody recognition test is associated with absolute pitch ability. *Scientific Reports*, *9*, Article 7838. https://doi.org/10.1038/s41598-019-44297-x

*West Marvin, E., & Brinkman, A. R. (2000). The effect of key color and timbre on absolute pitch recognition in musical contexts. *Music Perception*, *18*, 111-137. https://doi.org/10.2307/40285905

*West Marvin, E., VanderStel, J., & Siu, J. C.-S. (2020). In their own words: Analyzing the extents and origins of absolute pitch. *Psychology of Music*, *48*(6), 808-823. https://doi.org/10.1177/0305735619832959

Wickham, H., Averick, M., Bryan, J., Chang, W., McGowan, L. D., François, R., Grolemund, G., Hayes, A., Henry, L., Hester, J., Kuhn, M., Pedersen, T. L., Miller, E., Bache, S. M., Müller, K., Ooms, J., Robinson, D, Seidel D. P., … Yutani, H. (2019). Welcome to the tidyverse. *Journal of Open Source Software*, *4*, 1686. https://doi.org/10.21105/joss.01686

Wickham, H., Pedersen, T., & Seidel, D. (2022). *scales: Scale functions for visualization*. R package version 1.2.1, https://github.com/r-lib/scales, https://scales.r-lib.org

*Wilson, S. J., Lusher, D., Martin, C. L., Rayner, G., & McLachlan, N. (2012). Intersecting factors lead to absolute pitch acquisition that is maintained in a "fixed do" environment. *Music Perception*, *29*, 285-296. https://doi.org/10.1525/mp.2012.29.3.285

*Wilson, S. J., Lusher, D., Wan, C. Y., Dudgeon, P., & Reutens, D. C. (2009). The neurocognitive components of pitch processing: Insights from absolute pitch. *Cerebral Cortex*, *19*, 724-732. https://doi.org/10.1093/cercor/bhn121

*Wong, Y. K., & Wong, A. C. N. (2014). Absolute pitch memory: Its prevalence among musicians and dependence on the testing context. *Psychonomic Bulletin and Review*, *21*, 534-542. https://doi.org/10.3758/s13423-013-0487-z

*Wu, C., Kirk, I. J., Hamm, J. P., & Lim, V. K. (2008). The neural networks involved in pitch labeling of absolute pitch musicians. *NeuroReport*, *19*, 851-854. https://doi.org/10.1097/WNR.0b013e3282ff63b1

Zatorre, R. J. (2003). Absolute pitch: A model for understanding the influence of genes and development on neural and cognitive function. *Nature Neuroscience*, *6*, 692-695. https://doi.org/10.1038/nn1085

Zatorre, R. J., & Beckett, C. (1989). Multiple coding strategies in the retention of musical tones by possessors of absolute pitch. *Memory & Cognition*, *17*, 582-589. https://doi.org/10.3758/bf03197081

*Zatorre, R. J., Perry, D. W., Beckett, C. A., Westbury, C. F., & Evans, A. C. (1998). Functional anatomy of musical processing in listeners with absolute pitch and relative pitch. *Proceedings of the National Academy of Sciences of the United States of America*, *95*, 3172-3177. https://doi.org/10.1073/pnas.95.6.3172

*Ziv, N., & Radin, S. (2014). Absolute and relative pitch: Global versus local processing of chords. *Advances in Cognitive Psychology*, *10*, 15-25. https://doi.org/10.2478/v10053-008-0152-7

Supplementary Table 1

*AP Definitions*

| **Author** | **Definition of AP** |
| --- | --- |
| Akiva-Kabiri & Henik (2012) | People endowed with absolute pitch (AP) are able to identify single tones (i.e., pitch chroma) without any referential tonal context (p. 272) |
| Aruffo eg al. (2014) | the ability to identify individual sounds by their musical note names, such as "F-sharp" or "B-flat" (p. 186) |
| Athos et al. (2007) | the ability to name the pitch of a tone without the use of a reference tone (p. 14795) |
| Baharloo et al. (1998) | Absolute pitch (AP), also known as “perfect pitch,” refers to the ability to recognize the pitch of a musical tone, without an external reference pitch (p. 224) |
| Baharloo et al. (2000) | Absolute pitch (AP) is a behavioral trait that is defined as the ability to identify the pitch of tones in the absence of a reference pitch (p. 755) |
| Bahr et al. (2005) | Absolute pitch (AP) is the ability to label a heard tone without the use of comparison to a known referent (p. 58) |
| Bairnsfather, Osborne et al. (2022) | [AP possessors can] identify and label musical pitches in isolation, without using an external reference pitch (p. 1) |
| Bairnsfather, Ullén et al. (2022) | the uncommon ability to name isolated musical pitches without the use of a reference tone (p. 140) |
| Barnea et al. (1994) | Absolute pitch (AP) is the ability to name the pitch of a note without reference to any previously sounded note (recognition) or to sing a named note without reference to a previously sounded note (recall) (p. 29) |
| Behroozmand et al. (2014) | the ability to assign verbal labels to absolute pitches and to identify notes without first determining the note’s tonal relationship with another known note (p. 98) |
| Benassi-Werke et al. (2012) | Absolute pitch participants possess the capacity to associate verbal codes with notes (Zatorre et al., 1998), and they also have the ability to use long-term memory to recall tones without reverberating a pitch continuously to maintain it in short-term memory (p. 1169) |
| Benner et al. (2023) | the rare ability to immediately and effortlessly identify the pitch of any given tone without relying on external reference (p. 7045) |
| Bermudez & Zatorre (2009) | the ability to identify the names of musical pitches without reference to a standard (p. 89) |
| Bermudez, Lerch et al. (2009) | the ability to identify note names without aid of a reference tone possessed by a minority of musicians (p.1583) |
| Brancucci, di Nuzzo et al. (2009) | the ability to identify the correct label of any note in the absence of a reference or of a musical context (p. 2937) |
| Brancucci, Dipinto et al. (2009) | the ability to name the pitch of a tone without the use of any external reference (p. 1) |
| Brauchli et al. (2019) | the ability to identify and/or reproduce a musical pitch without the aid of a reference pitch (p. 241) |
| Brauchli et al. (2020) | the ability to identify a musical pitch without an external reference (p. 1) |
| Brown et al. (2002) | the ability to identify the pitch of an isolated tone (p. 163) |
| Brown et al. (2003) | none explicitly stated |
| Burkhard et al. (2019) | the ability to identify and label tones without reference to keyality (p.1) |
| Burkhard et al. (2020) | [AP possessors can] classify pitches absolutely into tone categories without the use of a reference tone (p. 1) |
| Burnham et al. (2015) | the ability to identify the chroma (pitch class) of a tone presented in isolation (e.g. middle C), or to produce a specified musical pitch without external reference (p. 883) |
| Burns & Campbell (1994) | The ability … to identify pure tones on the basis of frequency (p. 2706) |
| Chavarria-Soley (2016) | the unusual ability to recognize a pitch without an external reference (p. 891) |
| Coll et al. (2019) | the rare faculty to identify or produce instantaneously any musical tone or other sound according to the 12-tone equal temperament, in the strictest sense with semitone precision, without external reference (p. 1-2) |
| Crummer et al. (1994) | Persons with AP ability are able to identify a pitch without previous cuing, by relating the presented pitch to a long-term memory template or array, and conversely are able to correctly producea pitch on request (p. 2721) |
| Deutsch & Dooley (2013) | the ability to name or produce a note of particular pitch in the absence of a reference note (p. 1859) |
| Deutsch et al. (2006) | the ability to name a musical note in the absence of a reference note (p. 2398) |
| Deutsch et al. (2009) | the ability to name or produce a musical note of a given pitch in the absence of a reference note (p. 2829) |
| Deutsch et al. (2013) | the ability to name or produce a note of particular pitch in the absence of a reference note (p. 719) |
| Deutsch et al. (2021) | the ability to name or produce a note of given pitch in the absence of a reference note (p. 3853) |
| Di Giuseppe Germano et al. (2021) | the ability to identify any given pitch by name (accord- ing to the traditional pattern of musical notation learned by a subject), or by producing a given musical tone on demand, with no external reference, e.g., without a diapason (p. 2) |
| Dohn et al. (2012) | the ability to identify a musical pitch absolutely (i.e., without a reference pitch at hand) (p. 1379) |
| Dohn et al. (2014) | the ability to identify the pitch of a musical tone or to produce a musical tone at a given pitch without the use of an external reference pitch (p. 1) |
| Dohn et al. (2015) | the ability to identify the pitch of a musical tone or to produce a musical tone at a given pitch without the use of an external reference pitch (p. 359) |
| Dooley & Deutsch (2010) | the ability to name a musical note in the absence of a reference note (p. 890) |
| Dooley & Deutsch (2011) | the rare ability to name a musical tone in the absence of a reference tone (p. 4097) |
| Elmer et al. (2013) | The ability to identify a note without relying on a reference tone (p. 366) |
| Elmer et al. (2015) | the ability to either identify the pitch class (the chroma) of a tone or to produce a specific pitch without referring to a reference tone (p. 1736) |
| Fujisaki & Kashino (2002) | the ability to identify or produce a pitch of a sound without hearing a reference sound (p. 77) |
| Fujisaki & Kashino (2005) | identify or produce the pitch of a sound without hearing a reference sound (p. 315) |
| Greber & Jäncke (2020) | the unique ability to recognize the pitch class of a tone or to produce a specific pitch without the aid of a reference tone (p. 2) |
| Greber et al. (2018) | the rare ability to effortlessly identify the pitch of a musical tone without the aid of an external reference tone (p. 11) |
| Greber et al. (2020) | the rare ability to label the pitch class (chroma) of a sound without an external reference (p. 2) |
| Gregersen et al. (1999) | the capacity to recognize and name the pitch of a musical note or ambient sound without the use of a reference pitch and with a minimum of deliberation (p. 911) |
| Gregersen et al. (2001) | a relatively uncommon cognitive ability possessed by a minority of professional and amateur musicians, characterized by their ability to identify pitch names, or to recall specific pitches, without benefit of a reference pitch; this is usually accomplished in a relatively effortless and instantaneous fashion (p. 280) |
| Gruhn et al. (2018) | the rare ability to identify or produce a given pitch without a reference tone (p. 110) |
| Hantz et al. (1992) | long-term memory for pitch. It is usually manifested by the ability to name pitches without prompting or the use of a reference pitch, or the ability to produce a given pitch on request (p. 28). |
| Hantz et al. (1995) | long-term memory for pitch. It is usually manifested by the ability to name pitches without prompting or the use of a reference pitch, or the ability to produce a given pitch on request (p. 55) |
| Hantz et al. (1997) | the ability to name pitches without prompting or the use of a reference pitch, or the ability to produce a given pitch on request (p. 451-452) |
| Hedger et al. (2013) | the perceptual ability to name musical notes without the aid of a reference note (p. 1496) |
| Hirata et al. (1999) | a kind of special musical skill with which one can recognize or sing any note without musical cues (p. 999) |
| Hirose et al. (2002) | a unique cognitive modality characterized by the ability to identify any tones without using an external reference pitch (p. 247) |
| Hirose et al. (2004) | the ability to identify any tones without reference (labeling) (p. 1383) |
| Hirose et al. (2005) | the ability to identify any tone without reference (labeling) (p. 1775) |
| Hou et al. (2014) | [AP possessors can] label or identify music notes with high accuracy and fast speed without external reference (p. 2) |
| Hou et al. (2021) | individuals with absolute pitch (AP), who can label or identify individual musical notes with high accuracy and fast speed without the benefit of an external reference (p. 136) |
| Hou et al. (2023) | the ability to identify an individual musical note from a set of many notes but without the aid of external note references (p. 821) |
| Hsieh & Saberi (2007) | none explicitly stated |
| Hsieh & Saberi (2008a) | none explicitly stated |
| Hsieh & Saberi (2008b) | none explicitly stated |
| Hsieh & Saberi (2009) | the rare ability of some musicians in identifying the pitch of a musical note from long-term memory without an acoustic referent (p. 232) |
| Hsieh et al. (2022) | he ability to name and produce an isolated musical note without a referent (p. 2) |
| Hutchins et al. (2015) | the rare ability, found almost exclusively in musicians, to identify or produce specific musical pitches without reference to another musical pitch (p. 256) |
| Hutka & Alain (2015) | the ability to identify or produce a specific pitch without a reference pitch (p. 344) |
| Itoh et al. (2003) | to identify pitch names of arbitrary tones without being given a reference pitch (p. 465) |
| Itoh et al. (2005) | the ability to name pitches of arbitrary tones or to produce a tone with designated pitch without being given a reference tone (p. 760) |
| Jäncke et al. (2012) | to identify tones without relying on an external and internal reference tone. (p. 1447) |
| Jiang et al. (2020) | the ability to name or produce isolated musical notes in the absence of a reference note (p. 6) |
| Kamiyama et al. (2010) | Accurate absolute pitch should facilitate the retrieval of auditory categorical representations in the absence of playing the piano (p. 335) |
| Keenan et al. (2001) | the ability to identify the pitch of any tone in the absence of a musical context or reference tone (p. 1402) |
| Kim & Knösche (2016) | the ability to recognize and label the pitch chroma of a given tone without external reference (p. 3486) |
| Kim & Knösche (2017) | the ability to recognize the pitch of any given tonal sound without external reference (p. 3899) |
| Kim et al. (2017) | an ability to identify or produce the pitch of any tones without referring to another tone (p. 2) |
| Lee & Hung (2008) | the ability to name or produce the note of a particular musical pitch in the absence of a reference note (p. 3236) |
| Lee & Lee (2010) | "the ability to identify the frequency or musical name of a specific tone or, conversely, the ability to produce some designated frequency, frequency level, or musical pitch without comparing the tone with any objective reference tone" (Ward, 1999) - quoted (p. 481) |
| Lee et al. (2011) | the ability to produce or name a musical tone without a reference pitch (p. 526) |
| Leipold et al. (2021) | the ability to identify the pitch of a sound without an external reference (p. 1723-1724) |
| Leipold, Brauchli et al. (2019) | [AP musicians] effortlessly perceive, recognize, and name pitches in absolute terms and without the help of a reference pitch (p. 132) |
| Leipold, Greber et al. (2019) | the rare ability to name a tone without reference (p. 2497) |
| Leipold, Oderbolz et al. (2019) | the ability to effortlessly name the pitch of a sound without an ex-ternal reference sound (p. 21) |
| Leite et al. (2016) | to identify and name the pitch of a particular sound without any external reference (p. 1-2) |
| Leite Filho et al. (2023) | the ability to identify the pitch of a musical note without external reference (p. 1) |
| Li (2021) | the ability to name or identify a note of a given pitch in the absence of a reference note (p. 704) |
| Loui et al. (2011) | the ability to name the pitch class of any given tone without a reference (p. 1015) |
| Loui et al.(2012) | the ability to name musical pitches without a reference (p. 632) |
| Luders et al. (2004) | None explicitly stated |
| Maeshima et al. (2018) | the ability to label the pitch of tones without any reference tones (p. 1487) |
| Maggu et al. (2021) | a unique ability to name or produce a given pitch without a reference (p. 1) |
| Masataka (2011) | the ability to identify the frequency or musical name of a specific tone, or to identify a tone without comparing it with any objective reference tone (p. 1) |
| Matsuda et al. (2013) | a long-term internal representation of the pitch of tones in the musical scale. AP is typically manifested behaviorally as the ability to identify, by the name of the musical note, the pitch of any sound without reference to another sound, or by producinga given musical tone on demand, as well as the ability to precisely distinguish a non-scale note from a scale note (p. 155) |
| Matsuda et al. (2019) | the ability to identify pitch names of arbitrary musical tones without being given a reference pitch (p. 1) |
| McKetton et al. (2018) | the ability to identify or recreate a given note or collection of notes in the absence of a reference note (p. 2930) |
| McKetton et al. (2019) | the remarkable ability to discern the pitch of an acoustic stimulus in the absence of a reference (e.g., identify a note played on a piano when listening without any other visual or auditory cue) (p. 201) |
| McLachlan et al. (2013) | the ability to identify single pitches using musical note names without a reference pitch (p. 1615) |
| Miller & Clausen (1997) | the ability to recognise and name tones representing the pitch classification scheme common in a (musical) culture, independent of context (p. 4) |
| Miskiewicz & Rakowski (2012) | the ability to identify the musical pitch of a tone without the use of a reference pitch (p. 988) |
| Mito (2003) | the ability to perceive pitch classes without any reference tones (p. 18) |
| Miyazaki (1992) | an ability to identify the musical pitch categories of isolated tones without regard to tonal contexts (p. 271) |
| Miyazaki (1993) | No explicit definition. Closest: absolute pitch may be quite useful in musical practice, particularly when transcribing a heard musical passage into a musical notation or singing a complicated melody at first sight (p. 962) |
| Miyazaki (1995) | an exceptional ability in that its possessors can perceive pitch classes of tones presented in isolation very accurately without any reference tones given (p. 55) |
| Miyazaki (2004) | to name the musical of a tone presented alone, with no tonal context available (p. 413) |
| Miyazaki & Rakowski (2002) | an ability to name single pitches with no reference tone given and produce pitches corresponding to designated pitch names or musical note symbols (p. 1337) |
| Miyazaki et al. (2012) | the ability to identify the names of musical pitch (pitch classes or tone chromas) of isolated tones accurately and quickly without reference to an external standard (p. 3484) |
| Miyazaki et al. (2018) | an ability to name or produce a note of a given pitch in the absence of a reference (p. 135) |
| Ngan et al. (2023) | the ability to identify or produce the pitch of a tone without any external reference (p. 1) |
| Oechslin, Imfeld et al. (2010) | the ability to detect a pitch without any tonal reference (p. 1) |
| Oechslin, Meyer et al. (2010) | [AP possessors can] identify tones without the aid of any reference tone (p. 447) |
| Ohnishi et al. (2001) | the ability to identify the frequency or musical name of a specific tone, or to identify a tone without comparing it with any objective reference tone (p. 754) |
| Pantev et al. (1998) | [AP possessors can] perfectly map specific tone frequencies onto response choices (p. 813) |
| Parkinson et al. (2014) | the ability to perceive and identify exact musical notes (p. 1) |
| Peng et al. (2013) | The ability to label or produce musical tones in the absence of a reference tone (p. 449) |
| Rakowski & Rogowski (2007) | The ability of some musicians to permanently remember the exact values of musical pitch like C, C#, D... etc. (p. 251) |
| Rakowski & Rogowski (2011) | The ability to preserve in memory all 12 standards of musical pitch (chromas) (p. 816) |
| Reis et al.(2021) | the relatively rare ability to label a musical note without the aid of a reference note (p. 2) |
| Remijn et al. (2024) | the ability to identify or produce a tone’s pitch class without the use of an external reference pitch (p. 165) |
| Renninger et al. (2003) | the ability to name or produce a note without the use of a reference pitch (p. 357) |
| Rogenmoser et al. (2015) | the ability to effortlessly identify the chroma of a tone without the aid of any reference pitch (p. 122) |
| Rogenmoser, Arnicane et al. (2021) | the ability to identify the chroma (pitch class) of a tone or to produce a specific pitch without the aid of any reference tones (p. 623) |
| Rogenmoser, Li et al. (2021) | the ability to identify the chroma of a tone without the aid of any reference (p. 286) |
| Rogowski & Rakowski (2010) | the ability to identify the pitch chroma or the musical name of a tone without the use of a reference pitch (p. 332) |
| Ross et al. (2004) | [AP possessors are] capable of identifying quickly and accurately the absolute frequency of tonal stimuli (p. 522) |
| Ross et al.(2003) | [AP possessors are] capable of quickly and accurately labeling tonal stimuli on the basis of their fundamental frequencies (without the use of a reference tone) (p. 1793) |
| Schlaug et al. (1995) | [AP musicians identified by asking if they could] sing any tone without a reference tone and if they could name any tone that was given to them without a reference tone (p. 701) |
| Schlemmer et al. (2005) | the ability to name or produce tonal pitches without the use of an external reference such as the piano or tuning fork (p. 465) |
| Schulze et al. (2009) | the ability to identify any pitch of the Western musical scale without an external reference tone (p. 1) |
| Schulze et al. (2013) | the ability to identify any pitch of the Western musical scale without an external reference tone (p. 1579) |
| Sharma et al. (2019) | [AP musicians possess] linguistic tags for decoding pitch-classes into verbal codes or symbols without having to resort to an external reference pitch. This ability to name pitches without a referent is called absolute pitch (p. 2) |
| Sharma et al. (2023) | the ability to name pitch-classes within less than a second without a reference pitch (p. 6465) |
| Shigeno (1993) | [AP possessors can] identify or produce musical pitch without reference to any standard tones, just as they identify and produce speech sounds (pp. 683) |
| Temperley & West Marvin (2008) | [AP possessors] are able to identify pitches (and therefore keys) in absolute terms (p. 199) |
| Tervaniemi et al. (1993) | the ability to identify or produce a tone, for example, in terms of Western musical scale without hearing a reference tone (p. 305) |
| Theusch & Gitschier (2011) | The rare pitch-naming ability (p. 173) |
| Theusch et al. (2009) | the rare ability to identify tones with their corresponding musical note names without the aid of a reference tone (p. 112) |
| Tseng & Hsieh (2024) | the relatively rare ability to identify isolated musical notes (p. 2) |
| Van Hedger & Nusbaum (2018) | the ability to name or produce any musical note without the aid of a reference note (p. 251) |
| Van Hedger et al. (2016) | the rare ability to name or produce any musical note without a reference note (p. 1268) |
| Van Hedger et al. (2018) | the ability to explicitly name orproduce a musical note without the aid of a reference note (p. 139) |
| Van Hedger et al. (2020) | the rare ability to name or produce any musical note without a reference note (p. 2) |
| Vanzella & Schellenberg (2010) | the ability to identify or produce a musical tone (e.g., middle C or concert A) without reference to an external standard (p. 1) |
| Vanzella et al. (2022) | the ability to effortlessly name or produce isolated pitches without the need for a reference pitch (p. 227) |
| Wayman et al. (1992) | an ability to utilize a long-term memory store for pitch. Subjects with this ability are able to retain a target pitch indefinitely without effort (p. 3527) |
| Weisman et al. (2012) | [AP possessors can] can accurately identify, classify, or produce the pitches of dozens of acoustic frequencies without an external referent (p. 46) |
| Weiss et al. (2015) | the ability to identify or produce isolated tones (e.g., middle C, concert A) without a reference tone (p. 867) |
| Wengenroth et al. (2014) | the rare auditory ability to immediately and effortlessly recognize or produce the pitch of any given tone without using an external reference (p. 1127) |
| Wenhart & Altenmüller (2019) | the ability to name or produce a musical tone without any reference (p. 1) |
| Wenhart, Bethlehem et al. (2019) | the ability to name or produce a musical tone without the use of a reference tone (p. 2) |
| Wenhart, Hwang et al. (2019) | the unique (<1%) ability to name or produce a musical tone without a reference (p. 2) |
| West Marvin et al. (2000) | "the ability to identify the pitch of a musical tone or to produce a musical tone at a given pitch without the use of an external reference pitch" (Takeuchi & Hulse, 1993, p. 345) (p. 111) |
| West Marvin et al. (2020) | the rare ability to identify or to produce a specific pitch without reference to an external standard (p. 808) |
| Wilson et al. (2009) | the ability to identify single pitches (p. 285) |
| Wilson et al. (2012) | the ability to identify a pitch (e.g., the musical note C) without using a reference tone (p. 724) |
| Wong & Wong (2014) | [AP possessors can] label or produce isolated tones accurately and effortlessly (p. 534) |
| Wu et al. (2008) | [AP possessors can] can immediately identify or produce a tone of particular pitch without the presence of a reference pitch (p. 851) |
| Zatorre et al. (1998) | a long-term internal representation for the pitch of tones in the musical scale. It is typically manifested behaviorally by the ability to identify, by the name of the musical note, the pitch of any sound without reference to another sound or by producing a given musical tone on demand (p. 3172) |
| Ziv & Radin (2014) | the ability to identify and name isolated tones without comparison to any reference pitch (p. 15) |

Supplementary Figure 1

*Search Strategy for (A) 2022 Search and (B) 2024 Search*

**(A)**

**(B)**

Supplementary Figure 2

*Smaller Publications Trees Showing Further Relationships Among Tasks*


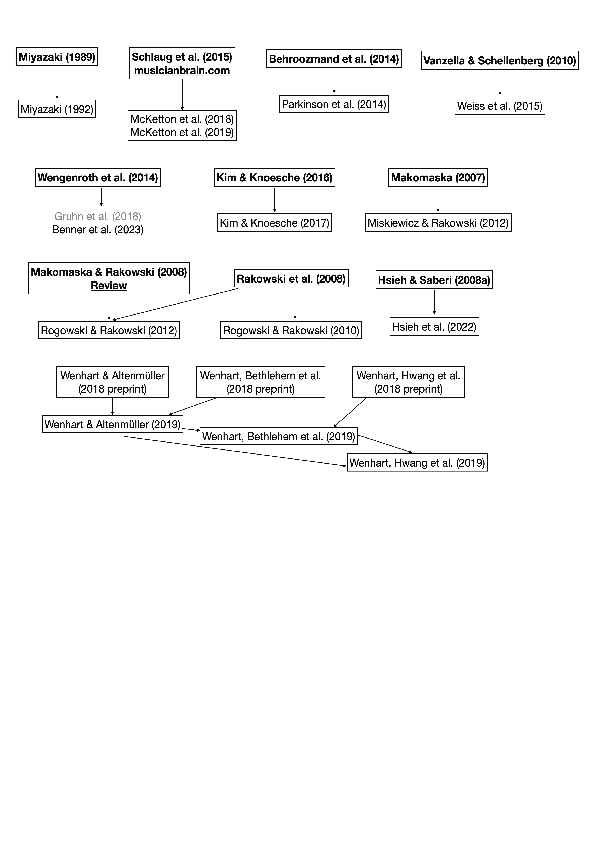

Supplement: Supplementary file 1 — Supplementary file1 (DOCX 3299 KB) [file 13428_2024_2577_MOESM1_ESM.docx]
